# Dysfunction of ventral tegmental area GABA neurons causes mania-like behavior

**DOI:** 10.1038/s41380-020-0810-9

**Published:** 2020-06-17

**Authors:** Xiao Yu, Wei Ba, Guangchao Zhao, Ying Ma, Edward C. Harding, Lu Yin, Dan Wang, Huiming Li, Peng Zhang, Youran Shi, Raquel Yustos, Alexei L. Vyssotski, Hailong Dong, Nicholas P. Franks, William Wisden

**Affiliations:** 1 Department of Life Sciences, Imperial College London, London, UK; 2 Department of Anesthesiology & Perioperative Medicine, Xijing Hospital, Xi’an, Shanxi China; 3 Institute of Neuroinformatics, University of Zürich/ETH Zürich, Zurich, Switzerland; 4 UK Dementia Research Institute, Imperial College London, London, UK

**Keywords:** Bipolar disorder, Neuroscience

## Abstract

The ventral tegmental area (VTA), an important source of dopamine, regulates goal- and reward-directed and social behaviors, wakefulness, and sleep. Hyperactivation of dopamine neurons generates behavioral pathologies. But any roles of non-dopamine VTA neurons in psychiatric illness have been little explored. Lesioning or chemogenetically inhibiting VTA GABAergic (VTA^*Vgat*^) neurons generated persistent wakefulness with mania-like qualities: locomotor activity was increased; sensitivity to D-amphetamine was heightened; immobility times decreased on the tail suspension and forced swim tests; and sucrose preference increased. Furthermore, after sleep deprivation, mice with lesioned VTA^*Vgat*^ neurons did not catch up on lost sleep, even though they were starting from a sleep-deprived baseline, suggesting that sleep homeostasis was bypassed. The mania-like behaviors, including the sleep loss, were reversed by valproate, and re-emerged when treatment was stopped. Lithium salts and lamotrigine, however, had no effect. Low doses of diazepam partially reduced the hyperlocomotion and fully recovered the immobility time during tail suspension. The mania like-behaviors mostly depended on dopamine, because giving D1/D2/D3 receptor antagonists reduced these behaviors, but also partially on VTA^*Vgat*^ projections to the lateral hypothalamus (LH). Optically or chemogenetically inhibiting VTA^*Vgat*^ terminals in the LH elevated locomotion and decreased immobility time during the tail suspension and forced swimming tests. VTA^*Vgat*^ neurons help set an animal’s (and perhaps human’s) mental and physical activity levels. Inputs inhibiting VTA^*Vgat*^ neurons intensify wakefulness (increased activity, enhanced alertness and motivation), qualities useful for acute survival. In the extreme, however, decreased or failed inhibition from VTA^*Vgat*^ neurons produces mania-like qualities (hyperactivity, hedonia, decreased sleep).

## Introduction

During the mania phase of bipolar disorder, patients sleep little and have elevated mood (e.g., increased energy and hyperactivity, impulsivity, decreased depression) [1–5]. In mice, pathological hyperactivity and elevated mood can be generated by various gene mutations and deletions: e.g., ClockΔ19 [6], REV-erbα [7], ErbB4 tyrosine kinase deletion in noradrenergic locus ceruleus cells [8], GSK-3β overexpression [9], dopamine transporter knockdown [10, 11], SHANK2 knockout [12], SHANK3 overexpression [13], ANK3 disruptions [14], ionotropic glutamate/AMPA receptor GluA1 knockout [15], ionotropic glutamate/kainate GluK2 knockout [16], phospholipase c γ1 knockout [17], histidine triad nucleotide-binding protein 1 knockout [18], glutamate-cysteine ligase modifier unit knockout [19], and the Na/K-ATPase α3 Myshkin (Myk/+) mutation [20–22]. Some of these gene manipulations alter excitation-inhibition (E-I) balance [8, 13, 15, 16, 23], and/or elevate catecholamines [6–8, 10, 24], suggesting common themes that could underlie the emergence of some types of mania.

Both these themes come together in the ventral tegmental area (VTA). The VTA an important source of dopamine, regulates goal- and reward-directed and social behaviors [25, 26], as well as wakefulness and sleep [27–29]. Exciting VTA dopamine neurons with well-chosen rhythms can produce mania-like behaviors in the day and euthymia at night [30]. In addition to dopamine neurons, there is a rich heterogeneity of glutamate and GABA neurons in the different anatomical subdomains of the VTA, and neurotransmitter co-release (e.g., dopamine -glutamate, GABA-glutamate, GABA-dopamine) from VTA neurons is common [25, 31–33].

For this paper, we focus on the midline VTA, which contains GABA (VTA^*Vgat*^) and glutamate/nitric oxide synthase (VTA^*Vglut2*^) neurons [25, 28, 34–37]. These VTA^*Vgat*^ and VTA^*Vglut2/NOS1*^ neurons inhibit and excite, respectively, the dopamine cells, but also, by projecting out of the VTA, exert effects independent of dopamine [25, 28, 36]. The VTA^*Vgat*^ neurons co-release GABA and glutamate [33], but the majority of these VTA neurons’ actions locally are GABAergic [28]. Locally they mostly inhibit VTA^*Vglut*2^, VTA^DA^ and VTA^*Vglut2*/DA^ cells, but also elicit a small number of pure excitatory responses [28]. Chemogenetic inhibition and chronic lesion of midline VTA^*Vgat*^ neurons causes sustained wakefulness [28, 29], and excitation of VTA^*Vglut2*^ cells and VTA dopamine cells also causes wakefulness [28, 27]. VTA^*Vgat*^ neurons limit arousal by inhibiting dopamine neurons and via projections to the lateral hypothalamus (LH) [28, 36, 38]; VTA^*Vglut2*^ neurons produce wakefulness, also independently of dopamine, by projecting to the LH and nucleus accumbens [28].

Here, we characterize the type of wakefulness produced by inhibiting or lesioning the VTA^*Vgat*^ neurons or exciting VTA^*Vglut2*^ neurons. We find that the wakefulness induced by diminishing or removing VTA^*Vgat*^ neuron inhibition contains behavioral endophenotypes that are mania-like, and are treatable with valproate and diazepam, although not with lithium or lamotrigine. On the other hand, the extended but quiet wakefulness produced by activating midline VTA^*Vglut2*^ neurons has no endophenotypes characteristic of mania. We suggest that the mania-like symptoms resulting from diminished VTA^*Vgat*^ function are generated by changing the E-I balance in both the VTA and the LH.

## Material and methods

### Mice and housing

All experiments were performed in accordance with the UK Home Office Animal Procedures Act (1986); all procedures were approved by the Imperial College Ethical Review Committee and the Ethics Committee for Animal Experimentation of Xijing Hospital, Xi’an, and were conducted according to the Guidelines for Animal Experimentation of the Chinese Council institutes. The following strains of mice were used: *Vgat-ires-Cre: Slc32a1*^*tm2(cre)Lowl*^/*J* kindly provided by Lowell, JAX stock 016962 [39]; *Vglut2-ires-Cre: Slc17a6*^*tm2(cre)Lowl*^*/J*, kindly provided by Lowell, JAX stock 016963 [39]. Mice were maintained on a 12 h:12 h light:dark cycle at constant temperature and humidity with ad libitum food and water. The “lights-on” period started at 6:00 p.m. (18:00); the “lights-off” period started at 6:00 a.m. (06:00). Lighting intensity was around 5 Lux during the red light “lights off period”; and the white light level around 150 Lux during the “lights on” period.

### AAV

*pAAV-hSyn-DIO-hM4Di-mCherry*, *pAAV-hSyn-DIO-hM3Dq-mCherry* and *pAAV-hSyn-DIO-mCherry* were gifts from Bryan L. Roth (Addgene plasmid 44362, 44361 and 50459) [40]; *pAAV-EF1α-DIO-taCASP3-TEV* was a gift from Nirao Shah (Addgene plasmid 45580) [41]; we packaged the transgenes into AAV capsids (capsid serotype 1/2) in house as described previously [42, 43]. *rAAV2/9-EF1a-DIO-eNpHR3.0-mCherry* and *rAAV2/9-EF1a-DIO-ChR2-mCherry* were packaged by BrainVTA (Wuhan, China).

### Surgery

10–12-week-old male mice were anesthetized with 2% isoflurane in oxygen by inhalation and received buprenorphine (0.1 mg/kg) and carprofen (5 mg/kg) injections, and then placed on a stereotaxic frame (Angle Two, Leica Microsystems, Milton Keynes, Buckinghamshire, UK). The AAV was injected through a stainless steel 33-gauge/15 mm/PST3 internal cannula (Hamilton) attached to a 10 µl Hamilton syringe, at a rate of 0.1 µl min^−1^. For the AAV injections, virus was bilaterally injected into the VTA, 50 nl for each side of the VTA. The injection co-ordinates were VTA: (ML = ±0.35 mm, AP = −3.52 mm, DV = −4.25 mm). After injection, the cannula was left at the injection site for 5 min and then slowly pulled out. After injections, mice that were to undergo the sleep experiments were implanted with three gold-plated miniature screw electrodes (–1.5 mm Bregma, +1.5 mm midline; +1.5 mm Bregma, –1.5 mm midline; –1 mm Lambda, 0 mm midline—reference electrode) with two EMG wire (AS634, Cooner Wire, CA). The EMG electrodes were inserted between the neck musculature. The Neurologger 2A EEG-EMG device [44] was affixed to the skull with Orthodontic Resin power and Orthodontic Resin liquid (Tocdental, UK). For the fiber optogenetic and chemogenetic experiments, after virus injection above the VTA (ML = ±0.35 mm; AP = −3.52 mm; DV = −4.25 mm), mice received surgical bilateral implantations above the LH of a monofiberoptic cannula (ML = ±0.36 mm; AP = −3.54 mm; DV = −4.0 mm) (200 µm; Doric Lenses, Inc., Quebec, Canada) or guide cannula (ML = ±0.36 mm; AP = −3.54 mm; DV = −3.5 mm) (World precision instruments, USA) for clozapine-N-oxide (CNO) delivery.

### Drug treatments

For all drug treatments, mice first received vehicle injections, and behavioral tests were performed; 2 weeks later, the same group of mice were given drug injections, and behavioral tests were performed. For the long-term valproate treatments, we further tested mice behaviors 2 weeks after valproate treatment was withdrawn. Baseline behaviors of the mice were tested during this period without any treatment.

### Chemogenetics

CNO (C0832, Sigma-Aldrich, dissolved in saline, 1 mg/kg) or saline was injected i.p. 30 min before the start of the behavioral tests. For VTA^*Vgat*^-hM4Di mice or VTA^*Vglut2*^-hM3Dq mice, CNO or saline was injected during the “lights off” active phase. 1 μl of 1 mM CNO was infused into the LH through the guide cannula at a rate of 0.1 µl min^−1^ with an injector needle (33-gauge, Hamilton)

### D-amphetamine

D-Amphetamine (2813/100, Tocris Bioscience, dissolved in saline, 2 mg/kg) or saline was injected i.p. into the mice [13, 21], and the mice were assessed directly after D-amphetamine injection.

### Valproate, lithium, diazepam, and lamotrigine

Sodium valproate (2815, TOCRIS, dissolved in saline, 200 mg/kg), LiCl (Sigma-Aldrich, dissolved in saline, 100 mg/kg) or saline (vehicle) was injected i.p. into the mice. For acute treatments (repeated injections) with lithium or valproate, mice received vehicle injections, and then behaviors (locomotion and tail-suspension) were tested; after 2 weeks, the same group of mice were given lithium or valproate injections. For the Li–H_2_O treatment, mice were treated with LiCl in drinking water (300 mg/L) for 2 weeks. Mice were first given normal water, and behaviors were tested, and the same group of mice were placed on Li–H_2_O for 2 weeks. Valproate or LiCl treatments were as previously reported [13, 17]: valproate or LiCl were injected three times (10:00, 14:00 and 17:00) one day before the behavioral assays; and during the day of the behavioral assay, valproate or LiCl was injected two times (10:00, 14:00). Locomotion or tail-suspension tests were performed 30 min after injection (14:30). After the locomotion or tail-suspension tests (see below), mice received a final valproate injection (17:00), and then the 24-h sleep–wake recordings were then performed (see section “EEG analysis, sleep–wake behavior and sleep deprivation”).

Diazepam (2805, TOCRIS, dissolved in 0.9% saline containing 0.1% Tween 80, 1 mg/kg) and lamotrigine (1611, TOCRIS, diluted in 0.9% saline, 10 mg/kg) or their control vehicles were injected i.p. into the mice. For single treatments with diazepam (1 mg/kg) or lamotrigine (10 mg/kg), mice received vehicle injections, and then behaviors (locomotion and tail-suspension) were tested; after 2 weeks, the same group of mice were given diazepam or lamotrigine injections (14:00). For repeated treatments, diazepam (1 mg/kg) or lamotrigine (10 mg/kg) were injected three times (10:00, 14:00, and 17:00) 1 day before the behavioral assays; and during the day of the behavioral assay, diazepam or lamotrigine were injected two times (10:00, 14:00). The locomotion or tail-suspension tests were performed 30 min after injection (14:30).

### Serum lithium

Blood samples were collected before and after lithium injections via transcutaneous cardiac puncture and the serum was separated for determination of lithium levels. Mice were maintained on anesthesia and blood samples were collected directly from the left ventricle before killing them. Serum lithium was analyzed using a Roche Cobas c311 analyzer.

### Dopamine receptor antagonists experiment

Dopamine antagonists SCH-23390 (0.03 mg/kg, dissolved in saline) and raclopride (1 mg/kg, dissolved in saline), for D1 and D2/D3 receptors, respectively, were injected serially i.p. into the VTA^*Vgat*^-mCherry mice or VTA^*Vgat*^-CASP3 mice 20 min before the locomotion, tail-suspension test (TST), or sleep experiment. For the experiments with chemogenetic inhibition combined with dopamine receptor antagonists, SCH-23390 and raclopride were injected serially i.p. into the VTA^*Vgat*^-hM4Di mice, and 20 min later, saline or CNO was injected into the antagonists-injected mice, and 30 min later, the locomotion test and TST was performed.

### Locomotor activity

The locomotor activity and time spent in stereotypy was detected in an activity test chamber (Med Associates, Inc) with an ANY-maze video tracking system (FUJIFILM co.) and measured by ANY-maze software (Stoelting Co. US.). VTA^*Vgat*^-mCherry or VTA^*Vgat*^-CASP3 mice were directly put into the activity test chamber; for VTA^*Vgat*^-hM4Di mice, the mice were put into the test chamber 30 min after saline or CNO injection; for D-Amphetamine experiments, mice were first put into chamber for 30 min, and the mice received vehicle or amphetamine injections. The locomotor activity was detected straight after injection for 1-h.

### Home cage activity

Mice were habituated in the cage for 24 h with mock Neurologgers before recordings. The home cage activities were then recorded using the accelerometer built into the Neurologger 2A devices [44] for a 24-h period and analyzed using Spike2 software.

### Tail-suspension test (TST)

The TST was performed as described [13]. After a habituation period in the test room, mice were suspended 60 cm above the floor by their tails by taping the tail tip. Behaviors were video recorded and blindly scored manually and measured by ANY-maze software (Stoelting Co. US).

### Forced swimming test (FST)

Mice were placed in a borosilicate glass cylinder (5 L, 18 cm diameter, 27 cm high) filled with water (25 °C, water depth 14 cm) for 6 min. The immobility time during the last 4 min was manually measured. Immobility time was defined as the time spent without any movements except for a single limb paddling to maintain flotation.

### Sucrose preference test (SPT)

This was a 2-choice test between 1% sucrose and water. Mice were habituated to a dual delivery system (one bottle with water and one bottle with 1% sucrose) for 3 days. Sucrose preference was then assessed over 3 consecutive days. For the chemogenetic experiments, animals were water-restricted overnight before saline or CNO injection. 30 min after saline or CNO injection, the mice were given free access to the 2-water delivery system for 4 h. Sucrose preference (%) was calculated as (weight of sucrose consumed)/(weight of water consumed + weight of sucrose consumed) × 100%.

### Elevated plus maze test

The elevated plus maze apparatus (Global Biotech Inc. Shanghai) was opaque and consisted of a central platform (10 cm × 10 cm), two open arms (50 cm × 10 cm), and two closed arms (50 cm × 10 cm) with protective walls 40 cm high, which was 70 cm above the ground. Animals were placed in the central platform facing one open arm of the apparatus and were free to explore the arms for 5 min. The apparatus was cleaned with 75% ethanol before and after each session. The traces were recorded by an overhead camera and shown by average heatmap of each group. The ratio of exploring time in the open arm was calculated by Video Tracking Software (ANY-maze, Stoelting Co., Ltd.).

### Timing of behavioral tests

10–12 weeks male mice were given virus injection (see “Surgery” section), and 4–6 weeks after this, behavioral tests were performed (the mice were ~14–18 weeks old by this stage), or drug treatments were started. During the drug treatment experiments, after vehicle injections, behavioral tests were performed (the mice were ~14–18 weeks old); and 2 weeks later, mice received drug injection, and behavioral tests were performed (by this stage, the mice were ~16–20 weeks old). All behavioral tests (locomotion, TST, forced swimming test (FST), or sucrose preference test (SPT)) took place during the “lights-off” phase of the light-dark cycle, when the mice were most active, particularly between 14:00 p.m.–17:00 (2 p.m.–5 p.m.), except for the sleep deprivation experiments (see section “EEG analysis, sleep–wake behavior, and sleep deprivation”).

### Mouse groupings for behavioral tests

For chemogenetic experiments, VTA^*Vgat*^-hM4Di mice were randomly split into two groups that received saline or CNO injection, and the locomotion test, TST, FST, or SPT were then performed. After 1–2 weeks, the same mice were given CNO or saline injection, and the locomotion test, TST, FST, or SPT were again performed. For the saline control experiments, VTA^*Vgat*^-hM4Di mice received saline injections, and then the locomotion test, TST, FST, or SPT were performed, and after 1–2 weeks, the same mice were given saline injection, and the locomotion test, TST, FST, or SPT were performed.

### EEG analysis, sleep–wake behavior, and sleep deprivation

EEG and EMG signals were recorded using Neurologger 2A devices [28, 44]. NREM sleep and wake states were automatically classified using a sleep analysis software Spike2 and then manually scored. The sleep deprivation protocol was as described previously [45]. At the start of “lights on” period, when sleep pressure is the highest, mice fitted with Neurologgers were put into novel cages, and at 1-h intervals, novel objects were introduced. After 5 h sleep deprivation, mice were then put back into their home cages for 19 h. In total, a 24-h sleep–wake state was recorded. For the sleep experiments with dopamine receptor antagonists, mice were given SCH-23390 (0.03 mg/kg) and raclopride (2 mg/kg) injections in the middle of the “lights off” active period.

### Optogenetic stimulation

For the optogenetic behavioral experiments, a fiber patch cord was connected to the laser generator, and dual optic fibers were connected to the fiber patch through a rotary joint (ThinkerTech Nanjing BioScience Inc. China.). Before the experiments, a monofiberoptic cannula was connected to the fiber patch cord. VTA^*Vgat*^-eNpHR-mCherry→LH mice or VTA^*Vgat*^-ChR2-mCherry→LH mice were bilaterally opto-stimulated (20 Hz, 0.5 s duration with 0.5 s interval, 593 nm, 20 μW) in the LH during the “lights off” phase. The stimulation was given for the duration of the behavioral tests.

### Immunohistochemistry

These procedures were carried as described previously [28]. Primary antibodies used were rat monoclonal mCherry (1:2000, Thermo Fisher, M11217); mouse monoclonal TH (1:2000, Sigma, T2928); secondary antibodies were Alexa Fluor 488 goat anti-mouse (1:1000, Invitrogen Molecular Probes, A11001), Alexa Fluor 594 goat anti-rat (1:1000, Invitrogen Molecular Probes, A11007). Slices were mounted on slides, embedded in Mowiol (with 4,6-diamidino-2-phenylindole), cover-slipped, and analyzed using an upright fluorescent microscope (Nikon Eclipse 80i, Nikon).

### Slice electrophysiology

Three weeks after injection of *AAV2/9-hSyn-DIO-hM3Dq-mCherry* into the VTA of *VGlut2-Cre* mice, brain slices containing the VTA were prepared for electrophysiological recordings. Mice were anesthetized with isoflurane and then killed. Brains were rapidly removed and placed in ice-cold oxygenated cutting ACSF (containing in mM: 252 sucrose, 2.5 KCl, 6 MgSO_4_, 0.5 CaCl_2_, 1.2 NaH_2_PO_4_, 2.5 NaHCO_3_, 10 glucose). Horizontal VTA slices (300 μm) were cut using a vibratome (VT1200S, Leica). Brain slices containing the VTA were transferred to recording ACSF with 124 mM NaCl, 2.5 mM KCl, 1.2 mM NaH_2_PO_4_, 2 mM MgSO_4_, 2 mM CaCl_2_, 24 mM NaHCO_3_, 5 mM HEPES and 12.5 mM glucose. Brain slices were incubated at 34 °C for 30 min and were then kept at room temperature under the same conditions for 45 min before transfer to the recording chamber at room temperature (22–25 °C). The ACSF was perfused at 2 mL/min. The brain slices were visualized with a fixed upright microscope (BX51WI, Olympus) equipped with a water immersion lens (40×/0.8 W) and a digital camera (C13440, Hamamatsu). Patch pipettes were pulled from borosilicate glass capillary tubes using a pipette puller (P97, Sutter). The resistance of pipettes varied between 3 and 5 MΩ. For current clamp recording of action potentials, pipettes were filled with solution (130 mM potassium gluconate, 4 mM KCl, 1 mM MgCl_2_, 4 mM ATP-Mg, 10 mM phosphocreatine, 0.3 mM EGTA, 0.3 mM GTP-Na, 10 mM HEPES, pH 7.3). The hM3Dq-mCherry-positive neurons in the VTA were patched in current-clamp mode. We injected a constant positive current to bring the membrane potential to around −45 mV to induce stable and persistent AP firing. CNO (100 μM) was bath applied. The signals were recorded with a MultiClamp 700B amplifier (Molecular Devices), Digidata 1550 interface, and Clampex 10.6 software (Molecular Devices).

### Quantification and statistics

Statistical tests were run in “Origin 2019b” (Origin Lab). The individual tests, and the number (*n*) of mice for each experimental group/condition, are given in the figure legends. All data are given as mean ± SEM and ‘center values’ are the means. We tested for normality and equal variances. When the data were non-normal, we used nonparametric tests (stated in relevant figure legends). Mice were assigned randomly to the experimental and control groups. For chemogenetic experiments, saline or CNO injection was blinded. For analyzing chronic lesioning experiments, VTA^*Vgat*^-mCherry and VTA^*Vgat*^-CASP3 mice, experimenters were blinded. For drug treatments, vehicle or drug injections were not done blinded. Experimental data analysis, including animal behavior that was scored from videos and the analysis of EEG data, was done blinded.

## Results

### Chemogenetic inhibition or lesioning of VTA^*Vgat*^ neurons produces increased locomotor activity and sensitivity to amphetamine

**Fig. 1:**
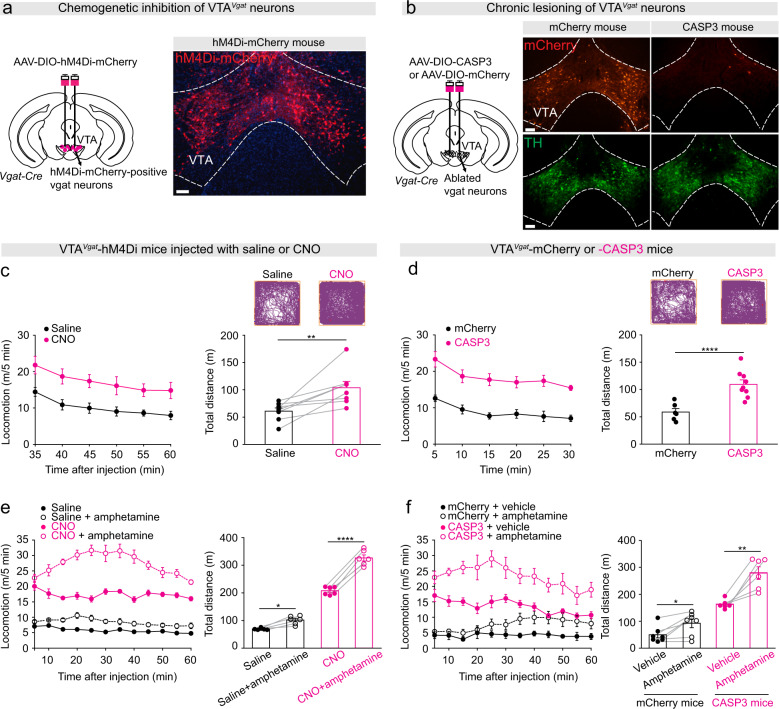
Selective inhibition or lesioning of VTA^*Vgat*^ neurons increased locomotor activity and sensitivity to D-amphetamine-induced hyperlocomotion. **a** Generation of VTA^*Vgat*^-hM4Di mice. Staining by immunohistochemistry (mCherry, red) for hM4Di-mCherry expression in the VTA. Scale bar: 150 μm. **b** Generation of VTA^*Vgat*^-CASP3 and VTA^*Vgat*^-mCherry control mice. Immunohistochemical staining for mCherry (red) and dopamine neurons (TH, tyrosine hydroxylase, green) in the VTA of control mice that expressed mCherry in VTA^Vgat^ neurons (left-hand images), or in mice where VTA^Vgat^ neurons were lesioned with caspase (right-hand images). Scale bar: 150 μm. **c** Video-tracked paths, locomotion speed and distance traveled of VTA^*Vgat*^-hM4Di mice (*n* = 8 mice) following CNO (1 mg/kg) injection compared with saline injection in the open field over a 30-min period. Paired *t*-test, *t* (7) = −3.68, **p = 0.007. **d** Video-tracked paths, locomotion speed and distance traveled of VTA^*Vgat*^-mCherry (*n* = 8 mice) and VTA^*Vgat*^-CASP3 mice (*n* = 9 mice) in the open field over a 30-min period. Unpaired *t*-test, t(15) = −5.43, ****p = 0.00006. **e** Locomotion speed and distance traveled of VTA^*Vgat*^*-*hM4Di mice (*n* = 6 mice) in the open field after saline or D-amphetamine injection (subsequent to saline or CNO injection). Repeated measures two-way ANOVA and Bonferroni-Holm post hoc test. *F*(1,5) = 66. Saline vs. saline + amphetamine *t*(5) = 3.69, *p = 0.01; CNO vs. CNO + amphetamine *t*(5) = 13.6, ****p = 0.000038. **f** Locomotion speed and distance traveled in the open field test of VTA^*Vgat*^*-*mCherry (*n* = 7 mice) or VTA^*Vgat*^-CASP3 mice (*n* = 6 mice) after vehicle or D-amphetamine injection. Two-way ANOVA and Bonferroni-Holm *post hoc* test. *F*_(mCherry or CASP3)_ = 93; *F*_(saline or amphetamine)_ = 26; *F*_(interaction)_ = 5.64; mCherry + vehicle vs. mCherry + amphetamine *t*(6) = −3.39, *p = 0.01; CASP3 + vehicle vs. CASP3 + amphetamine *t*(5) = −4.97, **p = 0.004. All error bars represent the SEM.

We delivered AAV-DIO-hM4Di-mCherry into the VTA of *Vgat-ires-cre* mice to express hM4Di-mCherry (an inhibitory receptor activated by the CNO ligand [40]) specifically in VTA^*Vgat*^ neurons to generate VTA^*Vgat*^-hM4Di mice (Fig. 1a) [28]. As previously [28], we also ablated VTA^*Vgat*^ neurons by injecting AAV-DIO-CASP3 and/or AAV-DIO-mCherry (as a control) into the VTA of *Vgat-ires-cre* mice to generate VTA^*Vgat*^-CASP3 mice and VTA^*Vgat*^-mCherry mice respectively. After 5–6 weeks post-injection, mCherry-positive VTA^*Vgat*^ neurons were detected in control mice that had only received AAV-DIO-mCherry injections, whereas mCherry-positive VTA^*Vgat*^ neurons were mostly absent in caspase-injected mice (Fig. 1b), but tyrosine hydroxylase-positive (dopamine) neurons were still present (Fig. 1b).

We next assessed locomotion behaviors of these animals. After CNO injection, VTA^*Vgat*^-hM4Di mice had higher locomotor activity in an open-field arena with more distance traveled (Fig. 1c) and had elevated average and maximum speeds over 30 min compared with saline-injected VTA^*Vgat*^-hM4Di mice (Supplementary Fig. 1a). As a control, the locomotor baseline activity of VTA^*Vgat*^-hM4Di mice did not differ over the extended experimental period: mice that received an initial saline injection, and then another saline injection two weeks later, had the same locomotor activity (Supplementary Fig. 1b). Consistent with these chemogenetic inhibition results, VTA^*Vgat*^-CASP3 mice also produced hyperlocomotion in the open field—more distance traveled (Fig. 1d), and higher average and maximum speeds over 30 min compared with the distance traveled by VTA^*Vgat*^-mCherry control mice (Fig. 1d; Supplementary Fig. 1c). VTA^*Vgat*^-CASP3 mice were also hyperactive in their home cages (Supplementary Fig. 1d). Notably, the hyperlocomotion of the VTA^*Vgat*^-CASP3 mice was still seen when the mice were retested in the open field four months post-lesion (Supplementary Fig. 1e). By the end of this period, the body weight of VTA^*Vgat*^-CASP3 mice was reduced compared with that of VTA^*Vgat*^-mCherry control mice (Supplementary Fig. 1f).

Mice suggested to have mania-like characteristics, or patients with bipolar-related mania, are hypersensitive to D-amphetamine [13, 21], whereas mice and patients posited to have an attention deficit hyperactivity (ADHD)-like disorder become less active with D-amphetamine [46, 47]. Treating CNO-injected VTA^*Vgat*^-hM4Di mice and VTA^*Vgat*^-CASP3 mice with D-amphetamine significantly increased their locomotion speed above their high baseline speed, and increased the distance traveled (Fig. 1e, f) and the time spent in stereotypy (Supplementary Fig. 1g, h), suggesting that both groups of mice (VTA^*Vgat*^-CASP3 and CNO-injected VTA^*Vgat*^-hM4Di mice) were not ADHD-like.

### Acute inhibition or chronic lesioning of VTA^*Vgat*^ neurons elevates mood

**Fig. 2:**
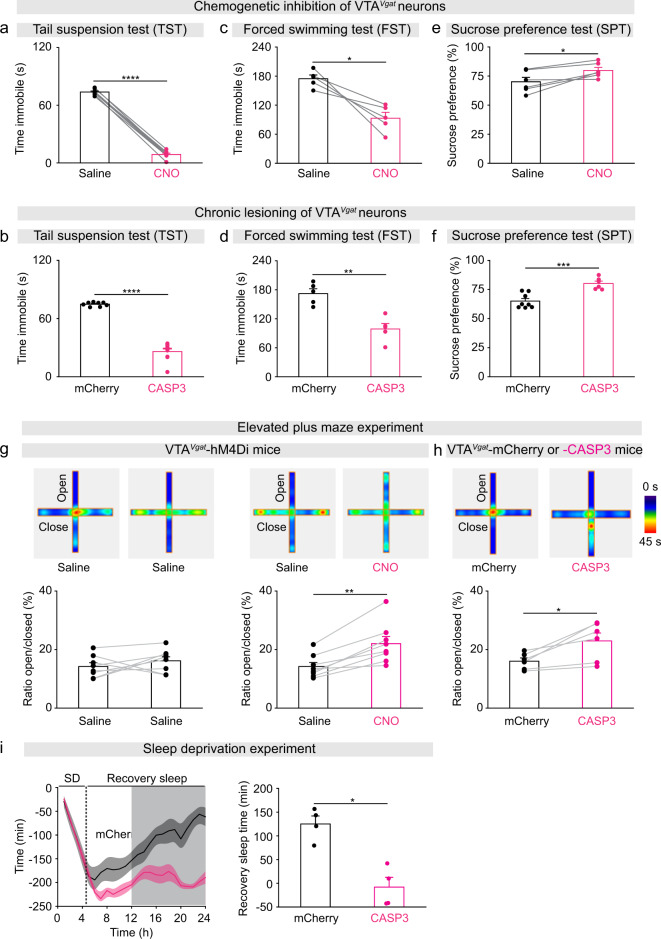
Dysfunction of VTA^*Vgat*^ neurons elevates mood and impairs sleep homeostasis. **a** Immobility time during the tail suspension test (TST) of VTA^*Vgat*^-hM4Di mice (*n* = 8 mice) after saline or CNO injection. Paired *t*-test, *t*(7) = 53.8, ****p = 1.98e^−10^. **b** Immobility time during the during TST of VTA^*Vgat*^-CASP3 (*n* = 9 mice) or VTA^*Vgat*^-mCherry mice (*n* = 8 mice). Unpaired *t*-test, t(15) = 14.3, ****p = 3.7e^−10^. **c** Immobility time during the forced swimming test (FST) of VTA^*Vgat*^-hM4Di mice (*n* = 6 mice) after saline or CNO injection. Paired *t*-test, *t*(5) = 5, **p = 0.003. **d** Immobility time during the FST of VTA^*Vgat*^-CASP3 (*n* = 6 mice) or VTA^*Vgat*^-mCherry mice (*n* = 6 mice). Unpaired t-test, *t*(10) = 6, ***p = 0.0001. **e** Sucrose preference of VTA^*Vgat*^-hM4Di mice (*n* = 6 mice) after saline or CNO injection. Paired *t*-test, *t*(5) = −3.5, *p = 0.01. **f** Sucrose preference of VTA^*Vgat*^-CASP3 (*n* = 6 mice) or VTA^*Vgat*^-mCherry mice (*n* = 8 mice). Unpaired *t*-test, *t*(12) = −4.8, ***p = 0.0004. **g** Elevated plus maze: VTA^*Vgat*^-hM4Di mice received saline injection and two weeks later a second saline injection (*n* = 8 mice), paired *t*-test, *t*(7) = −1.06, *p* = 0.32; or saline and 2 weeks later a CNO injection (*n* = 8 mice), paired *t*-test, *t*(7) = −5.28, **p = 0.001. **h** Elevated plus maze of VTA^*Vgat*^-CASP3 (*n* = 6 mice) or VTA^*Vgat*^-mCherry mice, paired *t*-test, *t*(5) = −3.45, *p = 0.01. **i** The accumulative recovery sleep (time in NREM sleep) after 5 h of sleep deprivation (SD) of VTA^*Vgat*^-CASP3 (*n* = 4 mice) or VTA^*Vgat*^-mCherry mice (*n* = 4 mice). Mann–Whitney test, *p = 0.03. The light and dark shading represents the “lights on” and “lights off” phases.

We assessed mood-related behaviors using TST, FST, SPT, and the elevated plus maze. During the TST, the immobility times of CNO-injected VTA^*Vgat*^-hM4Di mice and VTA^*Vgat*^-CASP3 mice were greatly decreased compared with saline-injected VTA^*Vgat*^-hM4Di control mice and VTA^*Vgat*^-mCherry (Fig. 2a, b). Both CNO-injected VTA^*Vgat*^-hM4Di mice and VTA^*Vgat*^-CASP3 mice also had decreased immobility times during the FST compared with saline-injected or VTA^*Vgat*^-mCherry controls (Fig. 2c, d). This could suggest that reducing VTA^*Vgat*^ neuronal output, by either chemogenetic inhibition or lesioning, produces less depressive-like behaviors. In addition, during the SPT, CNO-injected VTA^*Vgat*^-hM4Di mice and VTA^*Vgat*^-CASP3 mice consumed more sucrose than their control littermates (Fig. 2e, f), possibly indicating a raised hedonic state. As a control, the baseline behavior of VTA^*Vgat*^-hM4Di mice did not differ over the extended experimental period: mice that received an initial saline injection, and then another saline injection 2 weeks later, had the same performance during the TST, FST, or SPT (Supplementary Fig. 1i). We characterized the anxiety-like behavior of our mouse models using the elevated plus maze. The time spent in the open arms of the maze by both CNO-injected VTA^*Vgat*^-hM4Di mice and VTA^*Vgat*^-CASP3 mice increased (Fig. 2g, h), suggesting their anxiety was reduced.

### Mice with lesioned VTA^*Vgat*^ neurons have less sleep need after sleep deprivation

VTA^*Vgat*^-CASP3 mice have a long-term sleep deficit [28]. To further characterize the need of VTA^*Vgat*^-CASP3 mice for sleep, we performed a sleep deprivation experiment. After sleep deprivation, there is usually a rebound in lost NREM sleep, a process termed sleep homeostasis [48]. Mice were kept awake for 5 h with novel objects presented each hour and were then tested to see if they caught up on lost sleep in their home cage. Control VTA^*Vgat*^-mCherry mice had rebound NREM sleep after 5 h sleep deprivation, and thereby after 19 h they had regained 90% of their sleep loss (Fig. 2i). Surprisingly, the VTA^*Vgat*^-CASP3 mice, despite already starting from a chronic sleep-deprived baseline, did not catch up on lost NREM sleep after 5 h continuous sleep deprivation (Fig. 2i).

### The mania-like behavior produced by lesioning VTA^*Vgat*^ neurons can be pharmacologically rescued by valproate and diazepam but not lithium or lamotrigine

We assessed if lithium could treat the manic-like state of VTA^*Vgat*^-CASP3 mice. We confirmed that serum lithium was elevated to therapeutic levels during the acute treatment (Supplementary Fig. 2a), and that the behavioral baselines of VTA^*Vgat*^-mCherry or VTA^*Vgat*^-CASP3 mice did not change during the treatment period (Supplementary Fig. 2b–f). However, neither acute (100 mg/kg) (Supplementary Fig. 3a–e) nor chronic (300 mg/L) treatments (Supplementary Fig. 3f–j) had any effects (Of note, chronic Li-H_2_O treatment decreased the immobility time of control mice during the TST (Supplementary Fig. 3i)).

**Fig. 3:**
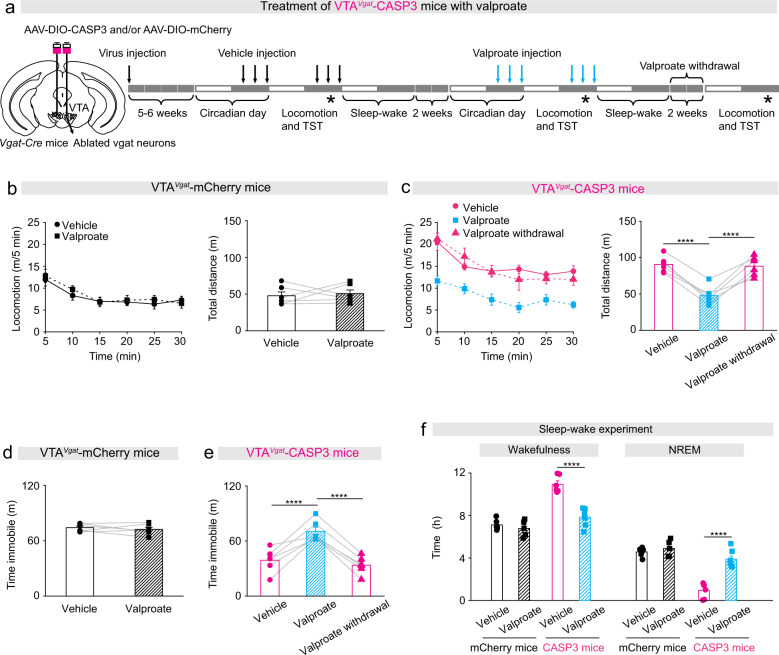
Valproic acid can successfully treat the mania-like behavior of VTA^*Vgat*^-CASP3 mice. **a** Pharmacological treatment protocol for valproic acid. The top arrows indicate vehicle or valproic acid injection (depending on mouse group) and the stars indicate when the behavioral experiments were undertaken. **b** Locomotion speed and distance traveled for VTA^*Vgat*^-mCherry mice (*n* = 6 mice) that received either vehicle or valproic acid treatment. Paired *t*-test, *t*(5) = −0.36, *p* = 0.73. **c** Locomotion speed and distance traveled for VTA^*Vgat*^*-*CASP3 mice (*n* = 6 mice) received either vehicle or valproic acid treatment, or where the valproic acid treatment had been removed for 2 weeks. Repeated measures one-way ANOVA and Bonferroni-Holm post hoc test. *F*(2,10) = 47; vehicle vs. valproate *t*(10) = 8.68, ****p = 0.000005; valproate vs. 2 weeks after valproate withdrawal *t*(10) = 8.19, ****p = 0.000009; vehicle vs. after valproate withdrawal *t*(10) = 0.48, *p* = 0.63. **d** Time spent immobile on the TST of VTA^*Vgat*^-mCherry mice (*n* = 6 mice) received vehicle or valproate injection. Paired *t*-test, *t*(5) = 0.71, *p* = 0.5. **e** Time spent immobile on the TST of VTA^*Vgat*^-CASP3 mice (*n* = 6 mice) received vehicle, valproate injection, or 2 weeks after valproate withdrawal. Repeated measures one-way ANOVA and Bonferroni-Holm post hoc test. *F*(2,10) = 32; vehicle vs. valproate *t*(10) = 7.41, ****p = 0.00002; 2 weeks after valproate withdrawal; *t*(10) = 6.32, ****p = 0.00008; vehicle vs. remove *t*(10) = 1.08, *p* = 0.3. **f** Percentage and time of wake and NREM for VTA^*Vgat*^-mCherry mice and VTA^*Vgat*^*-*CASP3 mice over the 12 h “lights off” period that received saline or valproate injection. mCherry + vehicle *n* = 6 mice; mCherry + valproate *n* = 5 mice; CASP3 + vehicle *n* = 6 mice; CASP3 + valproate *n* = 7 mice. Two-way ANOVA and Bonferroni-Holm post hoc test. Wake: *F*_(mCherry or CASP3)_ = 66; *F*_(Vehicle or valproate)_ = 17; *F*_(interaction)_ = 21; mCherry + vehicle vs. mCherry + valproate *t*(9) = 0.38, *p* = 0.38; CASP3 + vehicle vs. CASP3 + valproate *t*(11) = 6.89, ****p = 0.00002. NREM: *F*_(mCherry or CASP3)_ = 65; *F*_*(*Vehicle or valproate)_ = 32; *F*_(interaction)_ = 20; mCherry + vehicle vs. mCherry + valproate *t*(9) = −0.92, *p* = 0.37; CASP3 + vehicle vs. CASP3 + valproate *t*(11) = −6.85, ****p = 0.00002. All error bars represent the SEM.

We next examined valproate (200 mg/kg) treatments (Fig. 3a). Acute injection of valproate did not affect the locomotor activity of VTA^*Vgat*^-mCherry control mice (Fig. 3b). By contrast, the hyperlocomotor activity of VTA^*Vgat*^-CASP3 mice in the open field was restored down to control levels by valproate treatment (Fig. 3c). However, 2 weeks after the acute valproate treatment was withdrawal from VTA^*Vgat*^*-*CASP3 mice, their hyperactivity in the open field had returned (Fig. 3c). Similarly, during the tail vsuspension test, valproate treatment did not affect VTA^*Vgat*^-mCherry control mice (Fig. 3d), but significantly increased the immobility time of VTA^*Vgat*^-CASP3 mice back up to control levels (Fig. 3e). Two weeks after valproate had been removed, however, the abnormally high agitation of VTA^*Vgat*^-CASP3 mice had re-emerged (Fig. 3e).

We assessed if valproate could reduce the sustained wakefulness of VTA^*Vgat*^*-*CASP3 mice. The wake time of VTA^*Vgat*^*-*CASP3 mice significantly decreased after treatment with valproate, whereas NREM sleep time substantially increased (Fig. 3f). VTA^*Vgat*^-CASP3 mice also have a pathological sleep architecture with fewer episodes of wake and NREM sleep, prolonged duration of each wake episode, and substantially decreased numbers of wake-NREM transitions [28]. Valproate treatment normalized the sleep–wake architecture of the VTA^*Vgat*^-CASP3 mice: the episode number (Supplementary Fig. 4a), episode duration of wake was restored to control levels (Supplementary Fig. 4b), as were the number of transitions between wake and NREM sleep (Supplementary Fig. 4c).

We next tested if a low dose of the GABA_A_ receptor positive allosteric modulator diazepam, an anxiolytic, influenced the behavior of VTA^*Vgat*^*-*CASP3 mice (Supplementary Fig. 5a–j). Treatment of VTA^*Vgat*^-CASP3 mice with either single or repeated doses of 1 mg/kg diazepam partially reduced the hyperlocomotion (Supplementary Fig. 5c, h) and fully recovered the immobility time during the TST (Supplementary Fig. 5e, j). These results suggest that diazepam has a potential treatment effect on VTA^*Vgat*^*-*CASP3 mice.

We also treated VTA^*Vgat*^-CASP3 mice with another anti-epilepsy and bipolar disorder drug—lamotrigine (Supplementary Fig. 6a–j). Lamotrigine (10 mg/kg) decreased the locomotion (Supplementary Fig. 6b, g) and immobility time during the TST (Supplementary Fig. 6d, i) of VTA^*Vgat*^-mCherry mice. However, lamotrigine had no effect on treatment of VTA^*Vgat*^-CASP3 mice. Neither locomotion (Supplementary Fig. 6c, h) nor immobility time during TST (Supplementary Fig. 6e, j) were affected.

Note: with the exception of chronic lithium treatment, none of these drug treatments affected the overall body weight of the mice during and after treatments (Supplementary Fig. 7a–d); body weight was reduced during chronic lithium treatment (Supplementary Fig. 7c).

### The extended wakefulness produced by activating VTA^*Vglut2*^ neurons is not mania-like

**Fig. 4:**
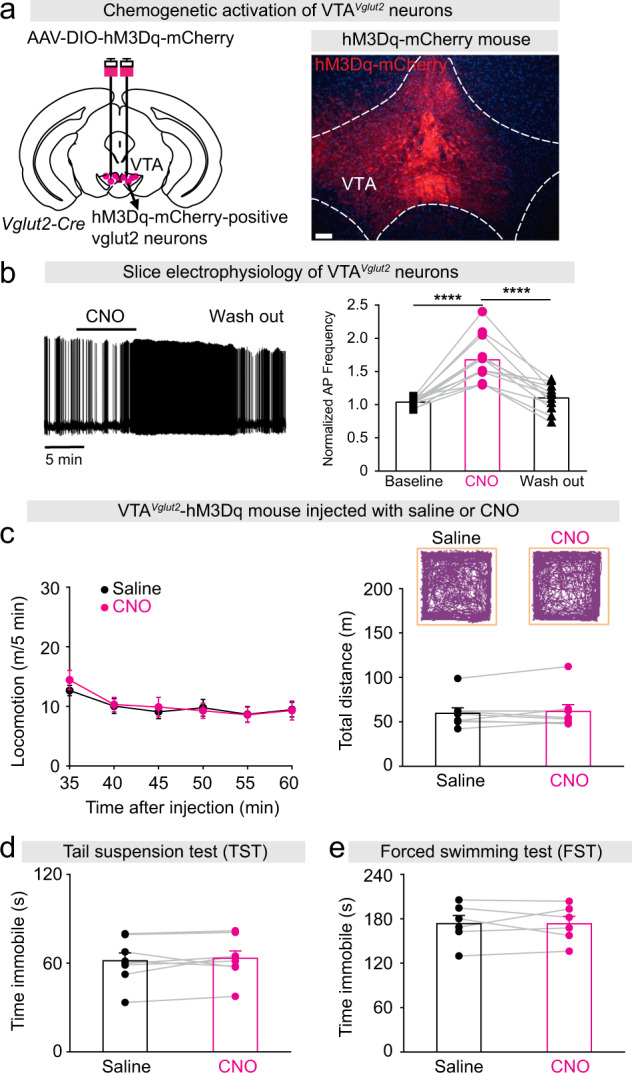
Activation of VTA^*Vglut2*^ neurons does not produce mania-like behaviors. **a** Generation of VTA^*Vglut2*^-hM3Dq mice. Staining by immunohistochemistry (mCherry) for hM3Dq-mCherry expression in the VTA. Scale bar: 150 μm. **b** Example of a spontaneously active VTA glutamatergic neuron with action potential firing before, during and after CNO application in an acute brain slice prepared from a VTA^*Vglut2*^-hM3Dq mouse. The bar graph shows the average normalized frequency of action potentials of all cells recorded. One-way repeated ANOVA and Bonferroni-Holm *post hoc* test. *F*_(baseline CNO washout) _= 35, baseline vs. CNO *t*(22) = 7.64, ****p = 1E-7, CNO vs. washout *t*(22) = 6.91, ****p = 6E-7, baseline vs. washout *t*(22) = 0.72, *p* = 0.4. **c** Video-tracked paths, locomotion speed and distance traveled of VTA^*Vglut2*^-hM3Dq mice (*n* = 8 mice) following CNO (1 mg/kg) injection compared with saline injection in the open field arena over a 30-min period. Paired *t*-test, *t*(7) = −0.72, *p* = 0.49. **d** Immobility time during the tail suspension test (TST) of VTA^*Vglut2*^-hM3Dq mice (*n* = 8 mice) after saline or CNO injection. Paired *t*-test, *t*(7) = −0.73, *p* = 0.88. **e** Immobility time during the forced swimming test (FST) of VTA^*Vglut2*^-hM3Dq mice (*n* = 6 mice) after saline or CNO injection. Paired *t*-test, *t*(4) = −0.01, *p* = 0.98.

Activating VTA^*Vglut2*^ neurons also promotes wakefulness [28]. To investigate if this type of artificially-induced wake is also mania-like, we expressed the excitatory chemogenetic hM3Dq receptor in VTA^*Vglut2*^ neurons (VTA^*Vglut2*^-hM3Dq mice; Fig. 4a) [8]. We prepared acute brain slices containing the VTA from VTA^*Vglut2*^-hM3Dq mice, and first confirmed that CNO application triggered action potentials in hM3Dq-mCherry-expressing cells in VTA glutamatergic neurons (Fig. 4b). Chemogenetic activation of VTA^*Vglut2*^ neurons by CNO injection did not alter the locomotor activity or distance traveled of VTA^*Vglut2*^-hM3Dq mice compared with saline injected mice (Fig. 4c) (see also data in 28). Moreover, the immobility time during the TST and FST did not differ between CNO injected- and saline injected VTA^*Vglut2*^-hM3Dq mice (Fig. 4d, e). This data serves as an internal negative control, showing that not all artificially induced wakefulness in mice will be mania-like: the activation of VTA^*Vglut2*^ neurons did not produce hyperactivity and mood-related deficits, suggesting a different kind of wakefulness from that generated by inhibition of VTA^*Vgat*^ neurons.

### VTA GABAergic neurons contribute to mania-like behaviors via dopamine signaling and projections to the LH

**Fig. 5:**
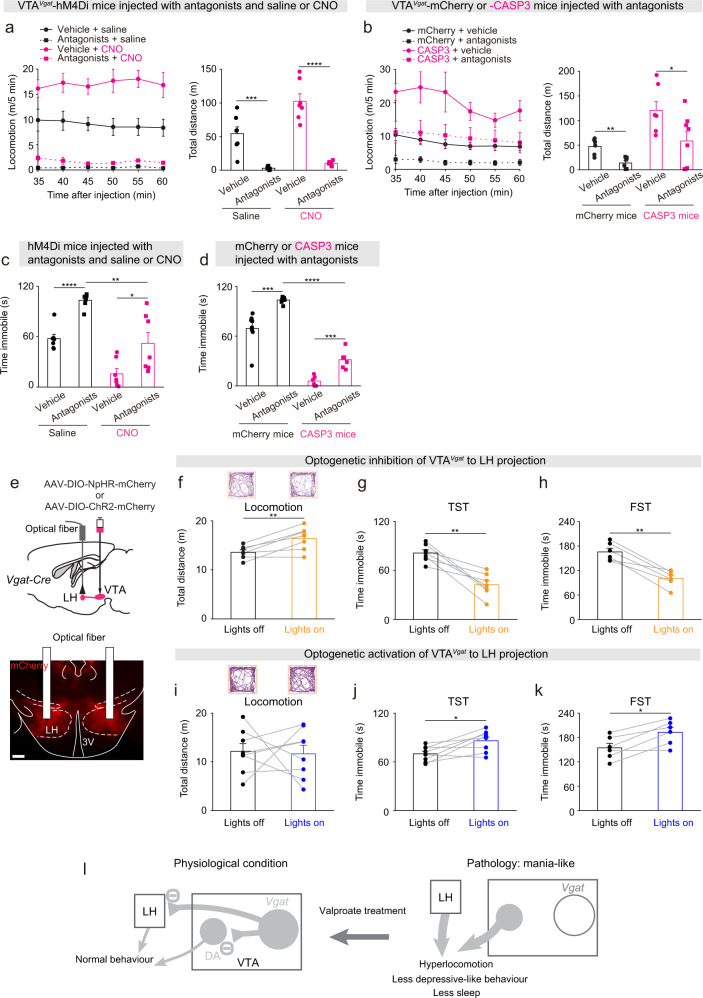
Circuit mechanisms underlying the mania-like behaviors mediated by VTA^*Vgat*^ neurons. **a** Locomotion speed and distance traveled of VTA^*Vgat*^-hM4Di mice that received injections with vehicle or a dopamine receptor antagonist mixture (SCH-23390 and raclopride for D1 and D2/D3 receptors, respectively) and either saline (*n* = 7 mice) or CNO (*n* = 7 mice). Two-way ANOVA and Bonferroni-Holm post hoc test. *F*_(saline or CNO)_ = 13.6; *F*_(vehicle or antagonists)_ = 91.2; *F*_(interaction)_ = 7.4; Vehicle + saline vs. antagonists + saline *t*(12) = 5, ***p = 0.0002; Vehicle + CNO vs. antagonists + CNO *t*(12) = 8.34, ****p = 2E−6. All error bars represent the SEM. **b** Locomotion speed and distance traveled of VTA^*Vgat*^-mCherry and VTA^*Vgat*^-CASP3 mice that received injections with either vehicle (*n* = 6 mice for mCherry and *n* = 10 mice for CASP3) or a dopamine receptor antagonist mixture (*n* = 6 mice for mCherry and *n* = 13 mice for CASP3). Two-way ANOVA and Bonferroni-Holm post hoc test. *F*_(mCherry or CASP3)_ = 10.8; *F*_(saline or antagonists)_ = 4.5; *F*_(interaction)_ = 0.03; mCherry + vehicle vs. mCherry + antagonists *t*(10) = 4.14, **p = 0.002; CASP3 + vehicle vs. CASP3 + antagonists *t*(21) = 1.64, *p* = 0.11. All error bars represent the SEM. **c** Time spent immobile for the tail suspension assay of VTA^*Vgat*^-hM4Di mice that received injections of either vehicle or the dopamine receptor antagonist mixture and saline or CNO. Two-way ANOVA and Bonferroni–Holm post hoc test. *F*_(saline or CNO)_ = 36; *F*_(Vehicle or antagonists)_ = 27; *F*_(interaction)_ = 0.35; vehicle + saline vs. antagonists + saline *t*(12) = −7.59, ****p = 6E−6; vehicle + CNO vs. antagonists + CNO *t*(12) = −2.54, *p = 0.02; antagonists + saline vs. antagonists + CNO *t*(12) = 3.8, **p = 0.002. All error bars represent the SEM. **d** Time spent immobile for the tail suspension assay of VTA^*Vgat*^-mCherry and VTA^*Vgat*^-CASP3 mice that received injections of either vehicle (*n* = 8 mice for mCherry and *n* = 9 mice for CASP3) or the dopamine receptor antagonist mixture (*n* = 8 mice for mCherry and *n* = 9 mice for CASP3). Two-way ANOVA and Bonferroni-Holm post hoc test. *F*_(mCherry or CASP3)_ = 209; *F*_(saline or antagonists)_ = 25; *F*_(interaction) _= 4.38; mCherry + vehicle vs. mCherry + antagonists *t*(14) = −4.87, ***p = 0.0002; CASP3 + vehicle vs. CASP3 + antagonists *t*(16)=−2.16, *p = 0.04; mCherry + antagonists vs. CASP3 + antagonists *t*(15) = 13, ****p = 7e^−10^. All error bars represent the SEM. **e** AAV-DIO-NpHR-mCherry was injected the VTA of *Vgat-ires-cre* mice, and optic fibers were bilaterally implanted above the LH. The NpHR-mCherry fibers projecting from the VTA^*Vgat*^ neurons into the LH were stained by immunohistochemistry (mCherry, red). Scale bar: 200 μm. **f** Video-tracked paths and distance traveled in the open field by VTA^*Vgat*^-NpHR mice (*n* = 7 mice) with and without opto-inhibition of VTA^*Vgat*^ terminals in the LH over 5 minute period. Paired *t*-test, *t*(6) = −4.08, **p = 0.006. **g** Time spent immobile on the tail suspension test (TST) of VTA^*Vgat*^-NpHR mice (*n* = 7 mice) with and without opto-inhibition of VTA^*Vgat*^ terminals in the LH. Paired *t*-test, *t*(6) = 5.3, **p = 0.001. **h** Time spent immobile on the forced swimming test (FST) of VTA^*Vgat*^-NpHR mice (*n* = 6 mice) with and without opto-inhibition of VTA^*Vgat*^ terminals in the LH. Paired t-test, *t*(5) = 6.77, **p = 0.001. **i** Video-tracked paths and distance traveled in the open field by VTA^*Vgat*^-ChR2 mice (*n* = 8 mice) with and without opto-activation of VTA^*Vgat*^ terminals in the LH over 5-minute period. Paired *t*-test, *t*(7) = 0.18, *p* = 0.85. **j** Time spent immobile on the tail suspension test (TST) of VTA^*Vgat*^-ChR2 mice (n = 8 mice) with and without opto-activation of VTA^*Vgat*^ terminals in the LH. Paired *t*-test, *t*(7) = −3.11, *p = 0.01. **k** Time spent immobile on the forced swimming test (FST) of VTA^*Vgat*^- ChR2 mice (*n* = 6 mice) with and without opto-activation of VTA^*Vgat*^ terminals in the LH. Paired *t*-test, *t*(5) = −4, *p = 0.01. **l** Conceptual circuit diagram illustrating VTA^*Vgat*^ neurons inhibiting VTA dopamine (DA) neurons and circuitry in the LH. When the VTA^*Vgat*^ cells have acutely diminished or absent function, activity of VTA dopamine neurons and arousal-promoting neurons in the LH increase. Valproate can reverse the effects of these changes.

To examine whether the hyperactive behaviors in the CNO-injected VTA^*Vgat*^-hM4Di mice or VTA^*Vgat*^-CASP3 mice were produced by increased dopamine signaling, we gave a dopamine receptor antagonist mixture i.p. (containing SCH-23390 and raclopride for D1 and D2/D3 receptors, respectively) (these antagonists were given prior to saline or CNO injection to VTA^*Vgat*^-hM4Di mice). In the open-field test, the dopamine antagonists reduced the hyperlocomotion and distance traveled of saline-injected VTA^*Vgat*^-hM4Di and VTA^*Vgat*^-mCherry control mice (Fig. 5a, b), and also largely reduced the hyperlocomotion of CNO-injected VTA^*Vgat*^-hM4Di mice (Fig. 5a), but only had a partial effect on VTA^*Vgat*^-CASP3 mice (Fig. 5b). In the TST, dopamine receptor antagonists increased the immobility time of both saline- or CNO-injected VTA^*Vgat*^-hM4Di mice and VTA^*Vgat*^-mCherry control or VTA^*Vgat*^-CASP3 mice (Fig. 5c, d). However, the immobility time of CNO-injected VTA^*Vgat*^-hM4Di mice or VTA^*Vgat*^-CASP3 mice that received dopamine receptor antagonist injection was still significantly lower than saline-injected VTA^*Vgat*^-hM4Di mice or VTA^*Vgat*^-mCherry mice injected with dopamine receptor antagonists (Fig. 5c, d). We also found that the extended wakefulness of VTA^*Vgat*^-CASP3 mice was substantially attenuated by the dopamine receptor antagonists (Supplementary Fig. 8a, b). The above results suggest that blocking dopamine signaling substantially reduces the mania-like behaviors of VTA^*Vgat*^-CASP3 mice.

VTA^*Vgat*^ neurons project prominently to the LH [28]. We conducted optogenetic and chemogenetic experiments to examine if the VTA^*Vgat*^ to LH projection contributes to hyperactivity and mood-related behaviors. An AAV carrying a Cre-dependent eNpHR3.0-mCherry transgene was injected into the VTA of *Vgat-ires-cre* mice to express inhibitory halorhodopsin in VTA^*Vgat*^ neurons to generate VTA^*Vgat*^-NpHR mice (Fig. 5e). Optic fibers were placed above the LH of VTA^*Vgat*^-NpHR mice, where dense NpHR-mCherry projections arising from the VTA^*Vgat*^ cell bodies can be seen (Fig. 5e). Optogenetic inhibition of the VTA^*Vgat*^ to LH projection, by activating NpHR-mCherry in the LH, elevated the locomotion of mice in the open field (Fig. 5f), whereas it decreased the immobility times during the TST (Fig. 5g) and FST (Fig. 5h). We confirmed this using specific chemogenetic inhibition of the VTA^*Vgat*^ to LH projection. CNO was infused directly into the LH of VTA^*Vgat*^-hM4D_i_ mice, which expressed the inhibitory CNO receptor in VTA^*Vgat*^ neurons (Supplementary Fig. 9a). CNO infusion increased locomotion speed (Supplementary Fig. 9b), and decreased immobility time during the TST (Supplementary Fig. 9c). These results indicate that inhibiting GABAergic tone from VTA^*Vgat*^ neurons to the LH produces hyperlocomotion and less depressive-like behaviors. We next injected AAV-DIO-ChR2-mCherry into the VTA of *Vgat-cre* mice to express excitatory channel rhodopsins in VTA GABAergic cells. Although opto-activation of the VTA^*Vgat*^ to LH projection did not affect the locomotion of mice in the open field (Fig. 5i), it did increase the immobility times during the TST (Fig. 5j) and FST (Fig. 5k). The above results suggest that the VTA^*Vgat*^ to LH projection, together with VTA^*Vgat*^ local inhibition of the dopamine system, contributes to generating mania-like behaviors.

## Discussion

We found that endophenotypes (hyperactivity, elevated mood, and reduced sleep) resembling aspects of mania could be produced by lesion or acute inhibition of GABAergic VTA^*Vgat*^ neurons. In mice with lesioned or inhibited VTA^*Vgat*^ neurons, D-amphetamine further increased the hyperactivity, suggesting the mania-like behavior of these mice could be the type associated with bipolar disorder in humans. The mania effects were partially blocked by D1, D2, and D3 receptor antagonists. Thus, hyperdopaminergia could originate from VTA^*Vgat*^ cells failing to inhibit dopamine neurons (Fig. 5l). VTA^*Vgat*^ cells, however, are not simply VTA inhibitory interneurons that reduce dopamine’s actions. Part of their actions in promoting increased movement in the FST and TST depends on their inhibitory projections to the LH. Our results on hyperactivity produced by disinhibiting LH circuits are consistent with previous findings. In rats, repeated delivery of subthreshold stimuli (kindling) to the LH induces mania-like behavior [49]. Indeed, the LH promotes physical activity and motivated behavior [50]; LH lesions in rodents and people produce a state of wakefulness with no motion [51]. The LH contains orexin/glutamate neurons that promote arousal, motivation and energy expenditure [52], and GABAergic neurons whose activation induces wakefulness and locomotion [50, 53, 54]. It is likely that the VTA^*Vgat*^ neurons inhibit both the orexin neurons and/or the arousal/locomotor- promoting GABA neurons in the LH [28, 36].

Given the hub-like nature of the GABAergic VTA^Vgat^ neurons, the mania phenotype produced by lesioning or inhibiting these neurons is likely a consequence of the disinhibition of multiple targets, e.g., dopamine cells in the VTA, but also to some extent other cell types in the LH. On the other hand, VTA^Vgat^ neurons also locally inhibit VTA glutamate neurons [28], but disinhibition of glutamate neurons in the VTA by lesioning or inhibiting the VTA^Vgat^ neurons probably does not contribute to the mania-phenotype. Direct pharmacogenetic excitation of these glutamate cells produces calm wakefulness (Fig. 4) [28].

Inability to sleep is one of the diagnostic criteria for the mania phase of bipolar disorder [2, 3, 55], and certainly VTA^*Vgat*^ mice sleep consistently less than control mice, such that they have 100% wakefulness during the “lights off” phase compared with control mice that take about 4 h NREM sleep during this time [28]. Usually, the longer that wakefulness persists, the stronger the urge to sleep becomes, until sleep is inescapable. A remarkable finding is that VTA^*Vgat*^-CASP3 mice bypassed the homeostatic process of having NREM recovery sleep after sleep deprivation—they did not catch up on lost sleep in spite of already starting from a strongly sleep-deprived background. The mechanisms underlying sleep homeostasis are not well understood [48, 56, 57]. Sleep homeostasis is thought to reflect the function of sleep, (otherwise why catch up on lost sleep?), and it will be interesting to investigate if the chronic lack of sleep of VTA^*Vgat*^-CASP mice will be detrimental metabolically.

Sleep deprivation can sometimes trigger mania episodes in humans [3, 55, 58]. Consequently, an interesting question is whether the chronic sleep deprivation phenotype of VTA^*Vgat*^-CASP3 mice actually causes their mania-like symptoms. Mice chronically sleep derived with the flower pot method—the animals stay on top of a raised platform surrounded by water, and when they fall asleep, they fall into water and wake up—also develop mania-like behavior [59]. This type of sleep deprivation is, however, likely to be stressful. In the VTA^*Vgat*^-CASP3 mice, the sleep-deprivation phenotype is internally generated within the brain, and could be less stressful *per se*.

The mania-like behaviors of VTA^*Vgat*^-CASP3 mice, including the strong sleep loss and abnormal sleep architecture, were reversed by valproate, and re-emerged when valproate treatment was stopped. Valproate has diverse actions: for example, it enhances GABAergic transmission and reduces action potential firing (reviewed in ref. 13), but by inhibiting histone deacetylases [60, 61], valproate treatments also change the expression of many genes. We found that low doses of diazepam were also efficacious in reducing the hyperarousal of VTA^*Vgat*^-CASP3 mice. Diazepam a GABA_A_ receptor positive allosteric modulator, enhances GABA transmission [62]. At the low doses tested here in mice, diazepam is an anxiolytic, but at higher doses (which we did not test) it induces sleep [62]. Two other important drugs used for treating bipolar disorder, lithium and lamotrigine, had no effect in treating VTA^*Vgat*^-CASP3 mice. Although its mechanism of action is unclear, lithium treatment is the first choice to treat mania episodes, although a subset of bipolar patients with rapidly cycling mania and depression phases are resistant to lithium (reviewed in ref 13). In most mouse models of mania, both valproate and lithium are usually effective treatments (reviewed in ref.^ 1^). But similar to our results, mice with SHANK3 overexpression in the neocortex, hippocampus and basal ganglia, have mania-like symptoms treatable with valproate but not lithium [13]. Similar arguments could apply to lamotrigine. It could be that the VTA^*Vgat*^-CASP3 mice and SHANK3-overexpressing mice models reflect a specific mania type.

In summary, the model based on reducing VTA^*Vgat*^ neuronal function could provide further insight into the genesis of some types of mania-like behaviors. One hypothesis is that VTA^*Vgat*^ neurons help set the level of mental and physical activity. Physiologically, inputs that inhibit VTA^*Vgat*^ neurons will transiently intensify aspects of wakefulness useful for acute success or survival: increased activity, enhanced alertness and motivation, reduced sleep (Fig. 5l). Taken to the extreme, however, pathology could emerge and decreased or failed inhibition from VTA^*Vgat*^ neurons will produce mania-like qualities (Fig. 5l).

## Supplementary information

Supplementary Material

## References

[1] Logan RW, McClung CA (2016). Animal models of bipolar mania: the past, present and future. Neuroscience.

[2] Gold AK, Sylvia LG (2016). The role of sleep in bipolar disorder. Nat Sci Sleep.

[3] Robillard R, Naismith SL, Hickie IB (2013). Recent advances in sleep-wake cycle and biological rhythms in bipolar disorder. Curr Psychiatry Rep.

[4] Harrison PJ, Geddes JR, Tunbridge EM (2018). The emerging neurobiology of bipolar disorder. Trends Neurosci.

[5] Phillips ML, Kupfer DJ (2013). Bipolar disorder diagnosis: challenges and future directions. Lancet.

[6] Roybal K, Theobold D, Graham A, DiNieri JA, Russo SJ, Krishnan V (2007). Mania-like behavior induced by disruption of CLOCK. Proc Natl Acad Sci USA.

[7] Chung S, Lee EJ, Yun S, Choe HK, Park SB, Son HJ (2014). Impact of circadian nuclear receptor REV-ERBalpha on midbrain dopamine production and mood regulation. Cell.

[8] Cao SX, Zhang Y, Hu XY, Hong B, Sun P, He HY (2018). ErbB4 deletion in noradrenergic neurons in the locus coeruleus induces mania-like behavior via elevated catecholamines. Elife.

[9] Prickaerts J, Moechars D, Cryns K, Lenaerts I, van Craenendonck H, Goris I (2006). Transgenic mice overexpressing glycogen synthase kinase 3beta: a putative model of hyperactivity and mania. J Neurosci.

[10] Young JW, Goey AK, Minassian A, Perry W, Paulus MP, Geyer MA (2010). The mania-like exploratory profile in genetic dopamine transporter mouse models is diminished in a familiar environment and reinstated by subthreshold psychostimulant administration. Pharm Biochem Behav.

[11] Young JW, van Enkhuizen J, Winstanley CA, Geyer MA (2011). Increased risk-taking behavior in dopamine transporter knockdown mice: further support for a mouse model of mania. J Psychopharmacol.

[12] Pappas AL, Bey AL, Wang X, Rossi M, Kim YH, Yan H (2017). Deficiency of Shank2 causes mania-like behavior that responds to mood stabilizers. JCI Insight.

[13] Han K, Holder JL, Schaaf CP, Lu H, Chen H, Kang H (2013). SHANK3 overexpression causes manic-like behaviour with unique pharmacogenetic properties. Nature.

[14] Leussis MP, Berry-Scott EM, Saito M, Jhuang H, de Haan G, Alkan O (2013). The ANK3 bipolar disorder gene regulates psychiatric-related behaviors that are modulated by lithium and stress. Biol Psychiatry.

[15] Maksimovic M, Vekovischeva OY, Aitta-aho T, Korpi ER (2014). Chronic treatment with mood-stabilizers attenuates abnormal hyperlocomotion of GluA1-subunit deficient mice. PLoS One.

[16] Shaltiel G, Maeng S, Malkesman O, Pearson B, Schloesser RJ, Tragon T (2008). Evidence for the involvement of the kainate receptor subunit GluR6 (GRIK2) in mediating behavioral displays related to behavioral symptoms of mania. Mol Psychiatry.

[17] Yang YR, Jung JH, Kim SJ, Hamada K, Suzuki A, Kim HJ (2017). Forebrain-specific ablation of phospholipase Cgamma1 causes manic-like behavior. Mol Psychiatry.

[18] Garzon-Nino J, Rodriguez-Munoz M, Cortes-Montero E, Sanchez-Blazquez P, Increased PKC (2017). activity and altered GSK3beta/NMDAR function drive behavior cycling in HINT1-deficient mice: bipolarity or opposing forces. Sci Rep.

[19] Kulak A, Cuenod M, Do KQ (2012). Behavioral phenotyping of glutathione-deficient mice: relevance to schizophrenia and bipolar disorder. Behav Brain Res.

[20] Timothy JWS, Klas N, Sanghani HR, Al-Mansouri T, Hughes ATL, Kirshenbaum GS (2017). Circadian disruptions in the Myshkin mouse model of mania are independent of deficits in suprachiasmatic molecular clock function. Biol Psychiatry.

[21] Kirshenbaum GS, Clapcote SJ, Duffy S, Burgess CR, Petersen J, Jarowek KJ (2011). Mania-like behavior induced by genetic dysfunction of the neuron-specific Na+,K+-ATPase alpha3 sodium pump. Proc Natl Acad Sci USA.

[22] Timothy JWS, Klas N, Sanghani HR, Al-Mansouri T, Hughes ATL, Kirshenbaum GS (2018). Circadian disruptions in the Myshkin mouse model of mania are independent of deficits in suprachiasmatic molecular clock function. Biol Psychiatry.

[23] Lee Y, Zhang Y, Kim S, Han K (2018). Excitatory and inhibitory synaptic dysfunction in mania: an emerging hypothesis from animal model studies. Exp Mol Med.

[24] Ashok AH, Marques TR, Jauhar S, Nour MM, Goodwin GM, Young AH (2017). The dopamine hypothesis of bipolar affective disorder: the state of the art and implications for treatment. Mol Psychiatry.

[25] Morales M, Margolis EB (2017). Ventral tegmental area: cellular heterogeneity, connectivity and behaviour. Nat Rev Neurosci.

[26] Russo SJ, Nestler EJ (2013). The brain reward circuitry in mood disorders. Nat Rev Neurosci.

[27] Eban-Rothschild A, Rothschild G, Giardino WJ, Jones JR, de Lecea L (2016). VTA dopaminergic neurons regulate ethologically relevant sleep-wake behaviors. Nat Neurosci.

[28] Yu X, Li W, Ma Y, Tossell K, Harris JJ, Harding EC (2019). GABA and glutamate neurons in the VTA regulate sleep and wakefulness. Nat Neurosci.

[29] Takata Y, Oishi Y, Zhou XZ, Hasegawa E, Takahashi K, Cherasse Y (2018). Sleep and wakefulness are controlled by ventral medial midbrain/pons GABAergic neurons in mice. J Neurosci.

[30] Sidor MM, Spencer SM, Dzirasa K, Parekh PK, Tye KM, Warden MR (2015). Daytime spikes in dopaminergic activity drive rapid mood-cycling in mice. Mol Psychiatry.

[31] Papathanou M, Creed M, Dorst MC, Bimpisidis Z, Dumas S, Pettersson H (2018). Targeting VGLUT2 in mature dopamine neurons decreases mesoaccumbal glutamatergic transmission and identifies a role for glutamate co-release in synaptic plasticity by increasing baseline AMPA/NMDA ratio. Front Neural Circuits.

[32] Pupe S, Wallen-Mackenzie A (2015). Cre-driven optogenetics in the heterogeneous genetic panorama of the VTA. Trends Neurosci.

[33] Root DH, Mejias-Aponte CA, Zhang S, Wang HL, Hoffman AF, Lupica CR (2014). Single rodent mesohabenular axons release glutamate and GABA. Nat Neurosci.

[34] Paul EJ, Kalk E, Tossell K, Irvine EE, Franks NP, Wisden W (2018). nNOS-expressing neurons in the ventral tegmental area and substantia nigra pars compacta. eNeuro.

[35] Taylor SR, Badurek S, Dileone RJ, Nashmi R, Minichiello L, Picciotto MR (2014). GABAergic and glutamatergic efferents of the mouse ventral tegmental area. J Comp Neurol.

[36] Chowdhury S, Matsubara T, Miyazaki T, Ono D, Fukatsu N, Abe M (2019). GABA neurons in the ventral tegmental area regulate non-rapid eye movement sleep in mice. Elife.

[37] Eban-Rothschild A, Borniger JC, Rothschild G, Giardino WJ, Morrow JG, de Lecea L (2020). Arousal-state dependent alterations in VTA-GABAergic neuronal activity. eNeuro.

[38] Yin L, Li L, Deng J, Wang D, Guo Y, Zhang X (2019). Optogenetic/chemogenetic activation of GABAergic neurons in the ventral tegmental area facilitates general anesthesia via projections to the lateral hypothalamus in mice. Front Neural Circuits.

[39] Vong L, Ye C, Yang Z, Choi B, Chua S, Lowell BB (2011). Leptin action on GABAergic neurons prevents obesity and reduces inhibitory tone to POMC neurons. Neuron.

[40] Krashes MJ, Koda S, Ye C, Rogan SC, Adams AC, Cusher DS (2011). Rapid, reversible activation of AgRP neurons drives feeding behavior in mice. J Clin Investig.

[41] Yang CF, Chiang MC, Gray DC, Prabhakaran M, Alvarado M, Juntti SA (2013). Sexually dimorphic neurons in the ventromedial hypothalamus govern mating in both sexes and aggression in males. Cell.

[42] Klugmann M, Symes CW, Leichtlein CB, Klaussner BK, Dunning J, Fong D (2005). AAV-mediated hippocampal expression of short and long Homer 1 proteins differentially affect cognition and seizure activity in adult rats. Mol Cell Neurosci.

[43] Murray AJ, Sauer JF, Riedel G, McClure C, Ansel L, Cheyne L (2011). Parvalbumin-positive CA1 interneurons are required for spatial working but not for reference memory. Nat Neurosci.

[44] Anisimov VN, Herbst JA, Abramchuk AN, Latanov AV, Hahnloser RH, Vyssotski AL (2014). Reconstruction of vocal interactions in a group of small songbirds. Nat Methods.

[45] Zhang Z, Ferretti V, Guntan I, Moro A, Steinberg EA, Ye Z (2015). Neuronal ensembles sufficient for recovery sleep and the sedative actions of alpha2 adrenergic agonists. Nat Neurosci.

[46] D’Andrea I, Fardella V, Fardella S, Pallante F, Ghigo A, Iacobucci R (2015). Lack of kinase-independent activity of PI3Kgamma in locus coeruleus induces ADHD symptoms through increased CREB signaling. EMBO Mol Med.

[47] Leo D, Gainetdinov RR (2013). Transgenic mouse models for ADHD. Cell Tissue Res.

[48] Porkka-Heiskanen T (2013). Sleep homeostasis. Curr Opin Neurobiol.

[49] Abulseoud OA, Camsari UM, Ruby CL, Mohamed K, Abdel Gawad NM, Kasasbeh A (2014). Lateral hypothalamic kindling induces manic-like behavior in rats: a novel animal model. Int J Bipolar Disord.

[50] Kosse C, Schone C, Bracey E, Burdakov D (2017). Orexin-driven GAD65 network of the lateral hypothalamus sets physical activity in mice. Proc Natl Acad Sci USA.

[51] Levitt DR, Teitelbaum P (1975). Somnolence, akinesia, and sensory activation of motivated behavior in the lateral hypothalamic syndrome. Proc Natl Acad Sci USA.

[52] Burdakov D (2018). Reactive and predictive homeostasis: Roles of orexin/hypocretin neurons. Neuropharmacology.

[53] Venner A, Anaclet C, Broadhurst RY, Saper CB, Fuller PM (2016). A novel population of wake-promoting GABAergic neurons in the ventral lateral hypothalamus. Curr Biol.

[54] Herrera CG, Cadavieco MC, Jego S, Ponomarenko A, Korotkova T, Adamantidis A (2016). Hypothalamic feedforward inhibition of thalamocortical network controls arousal and consciousness. Nat Neurosci.

[55] Wehr TA (1991). Sleep-loss as a possible mediator of diverse causes of mania. Br J Psychiatry.

[56] Greene RW, Bjorness TE, Suzuki A (2017). The adenosine-mediated, neuronal-glial, homeostatic sleep response. Curr Opin Neurobiol.

[57] Franken P (2013). A role for clock genes in sleep homeostasis. Curr Opin Neurobiol.

[58] Colombo C, Benedetti F, Barbini B, Campori E, Smeraldi E (1999). Rate of switch from depression into mania after therapeutic sleep deprivation in bipolar depression. Psychiatry Res.

[59] Benedetti F, Fresi F, Maccioni P, Smeraldi E (2008). Behavioural sensitization to repeated sleep deprivation in a mice model of mania. Behav Brain Res.

[60] Phiel CJ, Zhang F, Huang EY, Guenther MG, Lazar MA, Klein PS (2001). Histone deacetylase is a direct target of valproic acid, a potent anticonvulsant, mood stabilizer, and teratogen. J Biol Chem.

[61] Gottlicher M, Minucci S, Zhu P, Kramer OH, Schimpf A, Giavara S (2001). Valproic acid defines a novel class of HDAC inhibitors inducing differentiation of transformed cells. EMBO J.

[62] Wisden W, Yu X, Franks NP. GABA receptors and the pharmacology of sleep. Handbook of experimental pharmacology. CH-4052 Basal, Switzerland: Springer International Publishing AG; 2019. p. 279–304. 10.1007/164_2017_56 28993837

