# Supplementary material for: Dysfunction of ventral tegmental area GABA neurons causes mania-like behavior
Source: Mol Psychiatry. 2020 Jun 17;26(9):5213–28. doi: 10.1038/s41380-020-0810-9 (PMC8589652; doi:10.1038/s41380-020-0810-9)
Supplement: Supplementary file 1 — Supplementary Material [file 41380_2020_810_MOESM1_ESM.docx]

**Supplementary information**

**The supplementary information contains 9 figures and figure legends:**

**Supplementary Figure 1** (supports Figure 1 and 2): Chemogenetic inhibition or lesioning of VTA*^Vgat^* neurons produced increased locomotor activity and baseline controls

**Supplementary Figure 2** (supports Figure 3): Behavioral baseline during the treatment period and serum lithium level during treatment

**Supplementary Figure 3** (supports Figure 3): The mania-like behavior produced by lesioning VTA*^Vgat^* neurons cannot be treated by lithium

**Supplementary figure 4** (supports Figure 3f): Valproate treatment normalizes the sleep-wake architecture of the VTA*^Vgat^*-CASP3 mice

**Supplementary Figure 5** (supports Figure 3): The mania-like behavior produced by lesioning VTA*^Vgat^* neurons can be largely treated by diazepam

**Supplementary Figure 6** (supports Figure 3): The mania-like behavior produced by lesioning VTA*^Vgat^* neurons cannot be treated by lamotrigine

**Supplementary Figure 7** (supports Figure 3): Body weight of the mice during and after drug treatments

**Supplementary figure 8** (supports Figure 5): Blocking dopamine signaling restores extended wakefulness of VTA*^Vgat^*-CASP3 mice

**Supplementary figure 9** (supports Figure 5): Chemogenetic inhibition of the VTA*^Vgat^* to LH projection produces hyperlocomotion and less depressive-like behavior

**Supplementary figures**


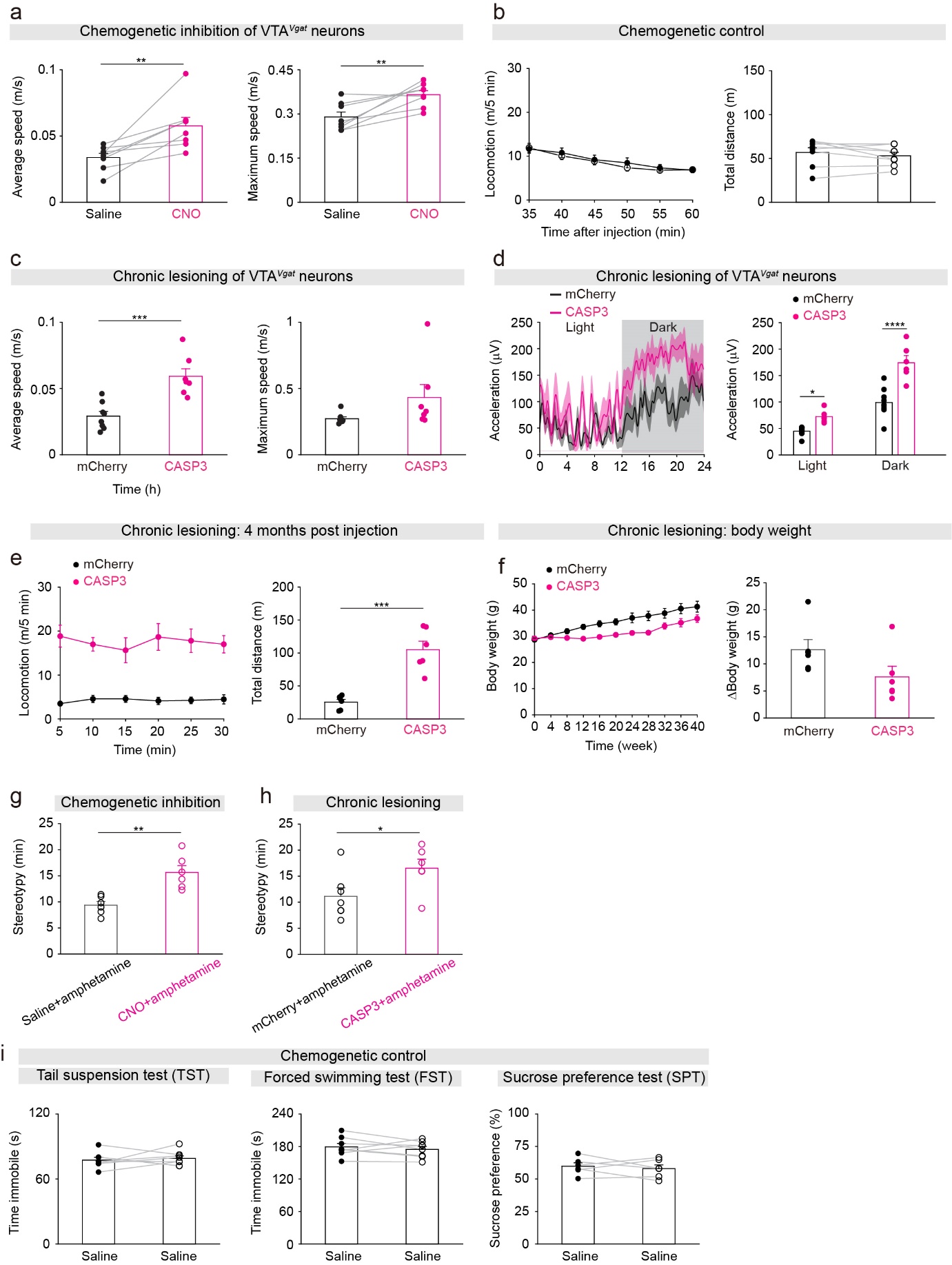


**Supplementary Figure 1 (supports Figure 1)**

**Chemogenetic inhibition or lesioning of VTA*^Vgat^* neurons produced increased locomotor activity; and baseline controls**

1. Average speed and maximum speed of VTA*^Vgat^*-hM4Di mice (n=8 mice) that received either saline or CNO injection. Paired t-test, average speed: t(7)=-4.57, **p=0.007; maximum speed: t(7)=-3.84, **p=0.006.
2. Behavioral baseline: Locomotion speed and distance travelled of VTA*^Vgat^*- hM4Di mice (n=8 mice) that received a saline injection and two weeks later a second saline injection. Unpaired t-test, t(7)=1.14, p=0.2.
3. Average speed and maximum speed of VTA*^Vgat^*-mCherry mice (n=8 mice) and control VTA*^Vgat^*-CASP3 mice (n=7 mice). Unpaired t-test, average speed: t(13)=-4.57, ***p=0.0005; maximum speed: t(13)=-1.72, p=0.1.
4. 24-h activity of VTA*^Vgat^*-mCherry mice (n=9 mice) and VTA*^Vgat^*-CASP3 mice (n=6 mice) in their home cages. Two-way repeated ANOVA and Bonferroni-Holm post hoc test. F_(light dark)_=119, light: mCherry vs. CASP3 t(13)=2.55, *p=0.02, dark: mCherry vs. CASP3 t(13)=7, ****p=8E-6,
5. 4 months post-AAV injection: locomotion speed and distance travelled of VTA*^Vgat^*-mCherry mice (n=6 mice) and VTA*^Vgat^*-CASP3 mice (n=6 mice). Unpaired t-test, t(10)=-5.8, ***p=0.0001.
6. Body weight development and change of body weight of VTA*^Vgat^*-mCherry mice (n=6 mice) and VTA*^Vgat^*-CASP3 mice (n=6 mice) Unpaired t-test, t(10)=1.85, p=0.09.
7. Time spent in stereotype of VTA*^Vgat^-*hM4Di mice (n=6 mice) in the open field after D-amphetamine injection (subsequent to saline or CNO injection). Unpaired t-test, t(10)=-4.2, **p=0.001.
8. Time spent in stereotype of VTA*^Vgat^-*mCherry mice (n=7 mice) and VTA*^Vgat^*-CASP3 mice (n=6 mice) in the open field after D-amphetamine injection. Unpaired t-test, t(11)=-2.24, *p=0.04.
9. Immobility time during the TST, FST or SPT of VTA*^Vgat^*-hM4Di mice (n=8 mice) after a saline injection and two weeks later a second saline injection. Paired t-test, TST: t(7)=-0.46, p=0.65; FST: t(7)=0.8, p=0.44; SPT: t(7)=1.14, p=0.28.


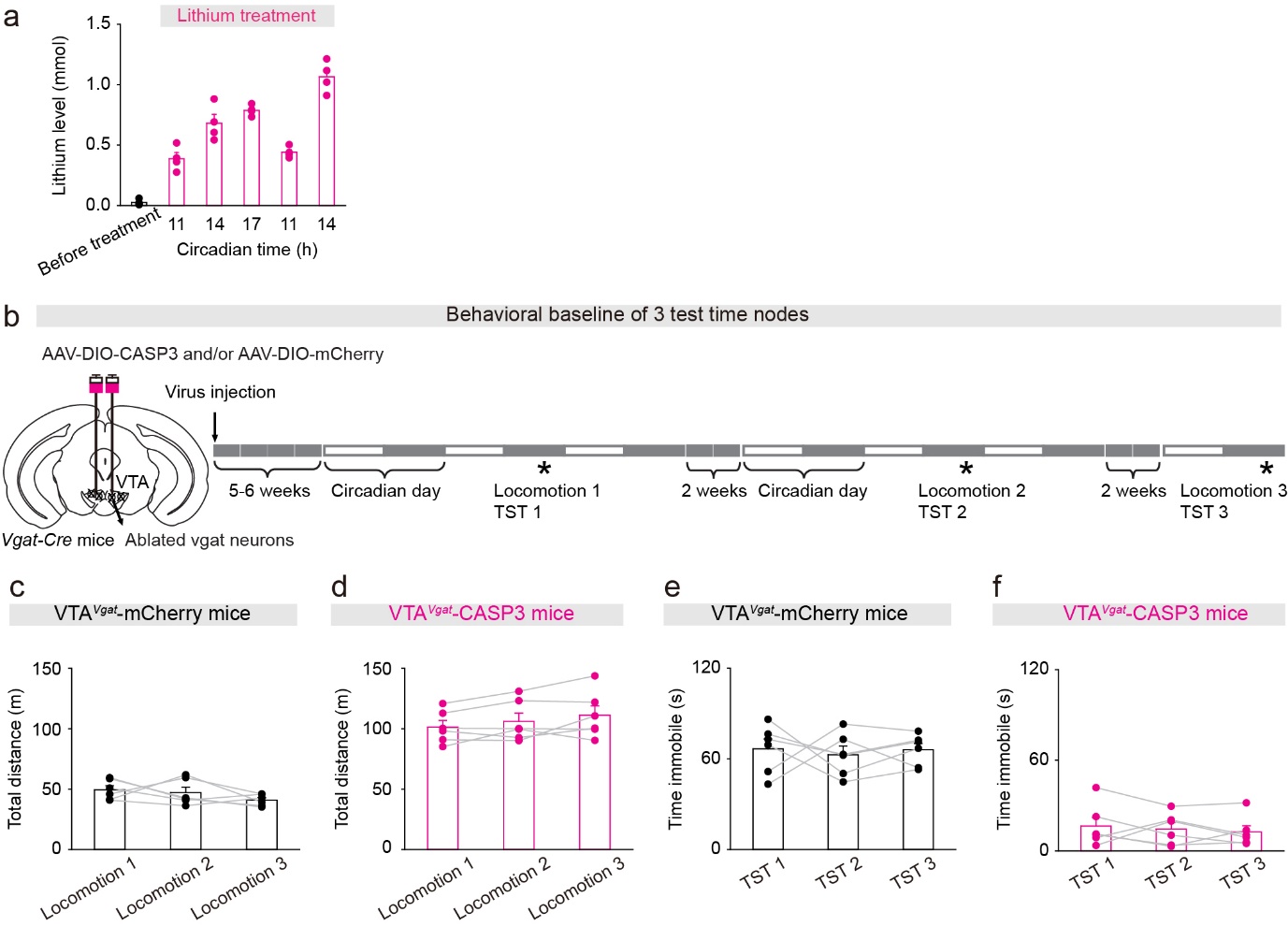


**Supplementary Figure 2** (supports Figure 3)

**Behavioral baseline during the treatment period and serum lithium level during the treatment**

1. Serum lithium level of VTA*^Vgat^*-CASP3 mice (n=4 mice for each time point) during the treatment.
2. Protocol for the measurement of baseline locomotor activity of VTA*^Vgat^*-mCherry mice and VTA*^Vgat^*-CASP3 mice. The stars indicate when the locomotion tests were undertaken.
3. Baseline locomotor activity of VTA*^Vgat^*-mCherry mice (n=6 mice). One-way repeated ANOVA and Bonferroni-Holm post hoc test. F_(locomotion1, 2, 3)_=1.3, locomotion 1 vs. locomotion 2 t(10)=0.44, p=0.66, locomotion 2 vs. locomotion 3 t(10)=1.17, p=0.26.
4. Baseline locomotor activity of VTA*^Vgat^*-CASP3 mice (n=6 mice). One-way repeated ANOVA and Bonferroni-Holm post hoc test. F_(locomotion1, 2, 3)_=3.1, locomotion 1 vs. locomotion 2 t(10)=1.22, p=0.25, locomotion 2 vs. locomotion 3 t(10)=1.29, p=0.22.
5. Baseline TST of VTA*^Vgat^*-mCherry mice (n=6 mice). One-way repeated ANOVA and Bonferroni-Holm post hoc test. F_(TST1, 2, 3)_=0.12, TST 1 vs. TST 2 t(10)=0.46, p=0.65, TST 2 vs. TST 3 t(10)=0.39, p=0.7.
6. Baseline TST of VTA*^Vgat^*-CASP3 mice (n=6 mice). One-way repeated ANOVA and Bonferroni-Holm post hoc test. F_(TST1, 2, 3)_=0.44, TST 1 vs. TST 2 t(10)=0.48, p=0.64, TST 2 vs. TST 3 t(10)=0.46, p=0.65.


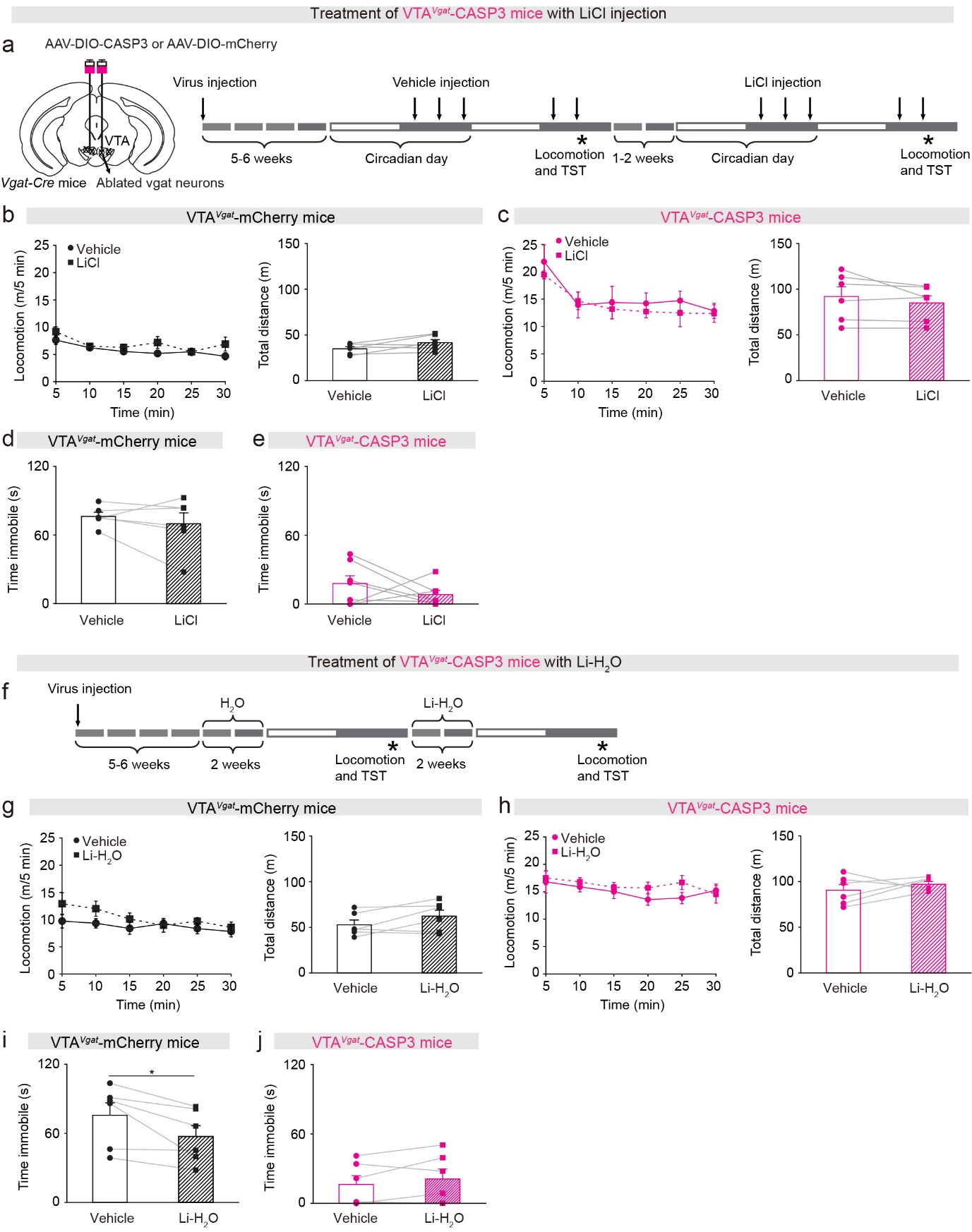


**Supplementary Figure 3 (supports Figure 3)**

**The mania-like behavior produced by lesioning VTA*^Vgat^* neurons cannot be treated by lithium**

(a) Pharmacological treatment protocol for LiCl. The top arrows indicate vehicle or LiCl injection (depending on mouse group) and the stars indicate when the behavioral experiments were undertaken.

(b) Locomotion speed and distance travelled of VTA*^Vgat^*-mCherry mice (n=6 mice) that received either vehicle or LiCl treatment. Paired t-test, t(5)=-2.08, p=0.09.

(c) Locomotion speed and distance travelled of VTA*^Vgat^-*CASP3 mice (n=6 mice) received either vehicle or LiCl treatment. Paired t-test, t(5)=1.39, p=0.22.

(d) Time spent immobile on the TST of VTA*^Vgat^*-mCherry mice (n=6 mice) that received either vehicle or LiCl injection. Paired t-test, t(5)=0.9, p=0.4.

(e) Time spent immobile on the TST of VTA*^Vgat^*-CASP3 mice (n=6 mice) that received either vehicle or LiCl treatment. Paired t-test, t(5)=1.08, p=0.32.

(f) Pharmacological treatment protocol for Li-H_2_O. The top arrows indicate that mice were feed with water or Li-H_2_O and the stars indicate when the behavioral experiments were undertaken.

1. Locomotion speed and distance travelled of VTA*^Vgat^*-mCherry mice (n=6 mice) that received either vehicle or Li-H_2_O treatment. Paired t-test, t(5)=-1.89, p=0.11.
2. Locomotion speed and distance travelled of VTA*^Vgat^-*CASP3 mice (n=6 mice) received either vehicle or Li-H_2_O treatment. Paired t-test, t(5)=-0.9, p=0.4.
3. Time spent immobile on the TST of VTA*^Vgat^*-mCherry mice (n=6 mice) that received either vehicle or Li-H_2_O treatment. Paired t-test, t(5)=2.85, p=0.03.
4. Time spent immobile on the TST of VTA*^Vgat^*-CASP3 mice (n=6 mice) that received either vehicle or Li-H_2_O treatment. Paired t-test, t(5)=-1.31, p=0.24.


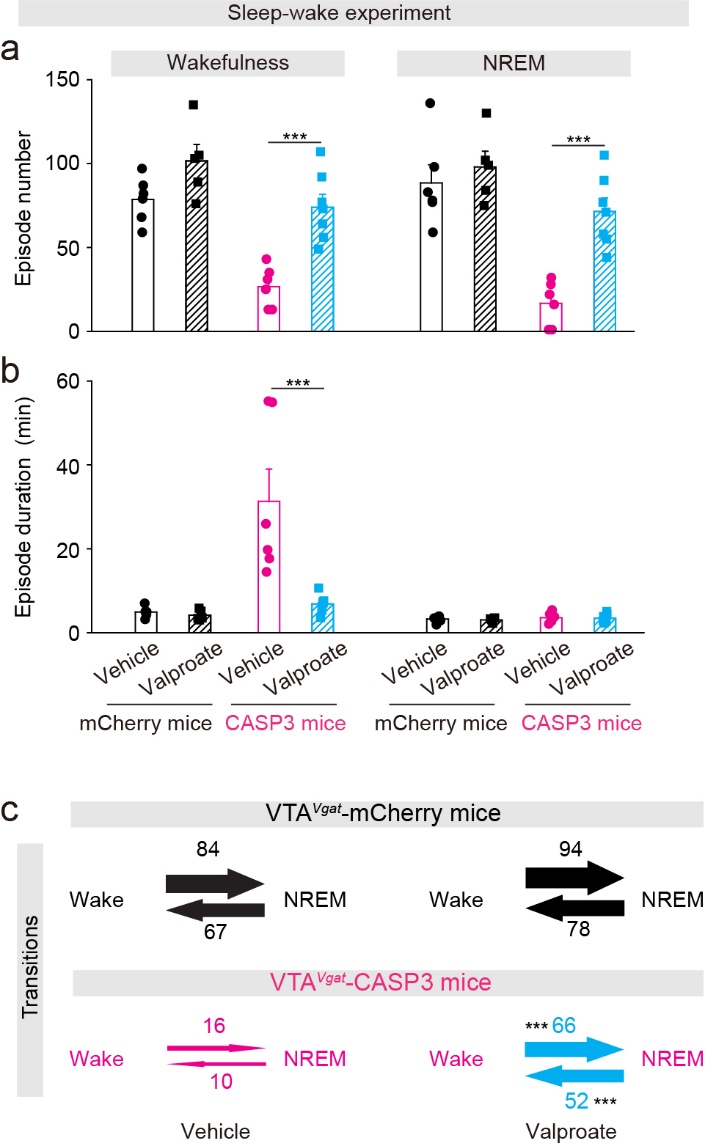


**Supplementary Figure 4 (supports Figure 3f)**

**Valproate treatment normalizes the sleep-wake architecture of the VTA*^Vgat^*-CASP3 mice**

1. Episode number of wake and NREM sleep for VTA*^Vgat^*-mCherry and VTA*^Vgat^*-CASP3 mice that received vehicle or valproate injection during the 12 hours “lights off” period. mCherry + vehicle n=6 mice; mCherry + valproate n=5 mice; CASP3 + vehicle n=6 mice; CASP3 + valproate n=7 mice. Two-way ANOVA and Bonferroni-Holm *post hoc* test. Wake: F_(mCherry or CASP3)_=30; F_(Vehicle or valproate)_=24; F_(interaction)_=2.9; mCherry + vehicle vs. mCherry + valproate t(9)=-2.12, p=0.06; CASP3 + vehicle vs. CASP3 + valproate t(11)=-4.98, ***p=0.0004. NREM: F_(mCherry or CASP3)_=32; F_(vehicle or valproate)_=13; F_(interaction)_=6; mCherry + vehicle vs. mCherry + valproate t(9)=-0.64, p=0.53; CASP3 + vehicle vs. CASP3 + valproate t(11)=-5.44, ***p=0.0002.
2. Episode duration of wake and NREM sleep for VTA*^Vgat^*-mCherry mice VTA*^Vgat^*-CASP3 mice that received vehicle or valproate injection. mCherry + Vehicle n=6 mice; mCherry + valproate n=5 mice; CASP3 + vehicle n=6 mice; CASP3 + valproate n=7 mice. Two-way ANOVA and Bonferroni-Holm *post hoc* test. Wake: F_(mCherry or CASP3)_=13; F_(vehicle or valproate)_=10; F_(interaction)_=9; mCherry + vehicle vs. mCherry + valproate t(9)=0.96, p=0.35; CASP3 + vehicle vs. CASP3 + valproate t(11)=3.45, **p=0.005. NREM: F_(mCherry or CASP3)_=0.86; F_(vehicle or valproate)_=0.23; F_(interaction)_=0.009; mCherry + vehicle vs. mCherry + valproate t(9)=0.55, p=0.59; CASP3 + vehicle vs. CASP3 + valproate t(11)=0.24, p=0.81.
3. Transitions of wake and NREM sleep for VTA*^Vgat^*-mCherry mice and VTA*^Vgat^*-CASP3 mice that received vehicle or valproate injection. mCherry + vehicle n=6 mice; mCherry + valproate n=5 mice; CASP3 + vehicle n=6 mice; CASP3 + valproate n=7 mice. Unpaired t-test, Wake to NREM: t(11)=-5.2, ***p=0.0002; NREM to wake: t(11)=-4.46, ***=0.0009. All error bars represent the SEM.


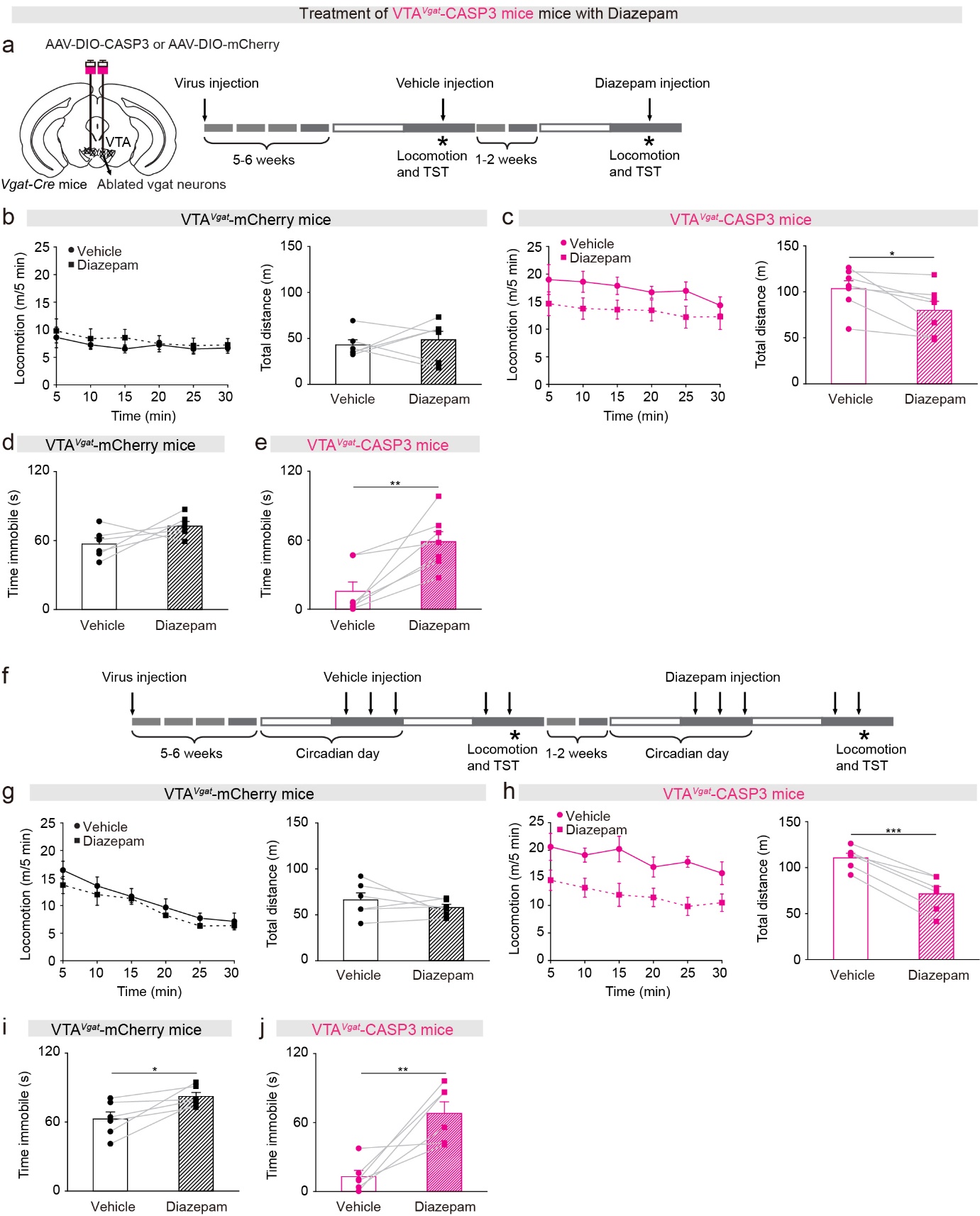


**Supplementary Figure 5 (supports Figure 3)**

**The mania-like behavior produced by lesioning VTA*^Vgat^* neurons can largely be treated by diazepam**

1. Pharmacological treatment protocol for diazepam ( 1 mg/kg). The top arrows indicate vehicle or diazepam injection (depending on mouse group) and the stars indicate when the behavioral experiments were undertaken.
2. Locomotion speed and distance travelled of VTA*^Vgat^*-mCherry mice (n=6 mice) that received either vehicle or diazepam treatment. Paired t-test, t(5)=-0.5, p=0.63.
3. Locomotion speed and distance travelled of VTA*^Vgat^-*CASP3 mice (n=7 mice) that received either vehicle or diazepam treatment. Paired t-test, t(6)=2.99, *p=0.02.
4. Time spent immobile on the TST of VTA*^Vgat^*-mCherry mice (n=6 mice) that received either vehicle or diazepam injection. Paired t-test, t(5)=-1.83, p=0.1.
5. Time spent immobile on the TST of VTA*^Vgat^*-CASP3 mice (n=7 mice) that received either vehicle or diazepam treatment. Paired t-test, t(6)=-3.94, **p=0.007.
6. Pharmacological repeated treatment protocol for diazepam. The top arrows indicate vehicle or diazepam injection and the stars indicate when the behavioral experiments were undertaken.
7. Locomotion speed and distance travelled of VTA*^Vgat^*-mCherry mice (n=6 mice) that received either vehicle or diazepam treatment. Paired t-test, t(5)=1.04 p=0.34.
8. Locomotion speed and distance travelled of VTA*^Vgat^-*CASP3 mice (n=6 mice) received either vehicle or diazepam treatment. Paired t-test, t(5)=10, ***p=0.0001.
9. Time spent immobile on the TST of VTA*^Vgat^*-mCherry mice (n=6 mice) that received either vehicle or diazepam treatment. Paired t-test, t(5)=-3, *p=0.02.
10. Time spent immobile on the TST of VTA*^Vgat^*-CASP3 mice (n=6 mice) that received either vehicle or diazepam treatment. Paired t-test, t(5)=-4.1, **p=0.008.


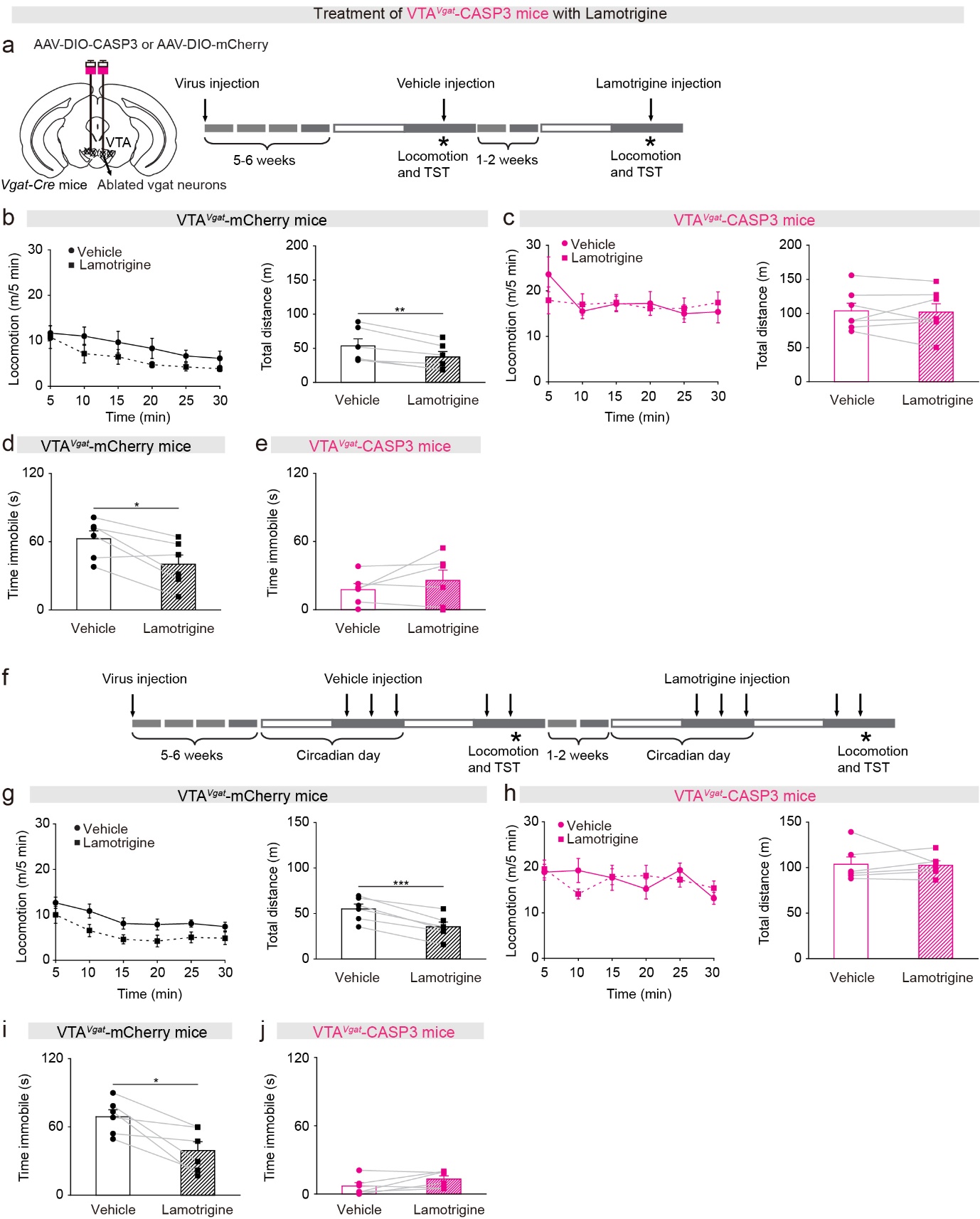


**Supplementary Figure 6 (supports Figure 3)**

**The mania-like behavior produced by lesioning VTA*^Vgat^* neurons cannot be treated by lamotrigine**

1. Pharmacological treatment protocol for lamotrigine (10 mg/kg). The top arrows indicate vehicle or lamotrigine injection (depending on mouse group) and the stars indicate when the behavioral experiments were undertaken.
2. Locomotion speed and distance travelled of VTA*^Vgat^*-mCherry mice (n=6 mice) that received either vehicle or lamotrigine treatment. Paired t-test, t(5)=5, **p=0.004.
3. Locomotion speed and distance travelled of VTA*^Vgat^-*CASP3 mice (n=7 mice) received either vehicle or lamotrigine treatment. Paired t-test, t(6)=0.24, p=0.8.
4. Time spent immobile on the TST of VTA*^Vgat^*-mCherry mice (n=6 mice) that received either vehicle or lamotrigine injection. Paired t-test, t(5)=3.31, *p=0.02.
5. Time spent immobile on the TST of VTA*^Vgat^*-CASP3 mice (n=6 mice) that received either vehicle or lamotrigine treatment. Paired t-test, t(5)=-1.22, p=0.27.
6. Pharmacological repeated treatment protocol for lamotrigine. The top arrows indicate vehicle or lamotrigine injection and the stars indicate when the behavioral experiments were undertaken.
7. Locomotion speed and distance travelled of VTA*^Vgat^*-mCherry mice (n=6 mice) that received either vehicle or lamotrigine treatment. Paired t-test, t(5)=7.7, ***p=0.0005.
8. Locomotion speed and distance travelled of VTA*^Vgat^-*CASP3 mice (n=6 mice) received either vehicle or lamotrigine treatment. Paired t-test, t(5)=0.17, p=0.87.
9. Time spent immobile on the TST of VTA*^Vgat^*-mCherry mice (n=6 mice) that received either vehicle or lamotrigine treatment. Paired t-test, t(5)=-3.59, *p=0.01.
10. Time spent immobile on the TST of VTA*^Vgat^*-CASP3 mice (n=6 mice) that received either vehicle or lamotrigine treatment. Paired t-test, t(5)=-1.88, p=0.1.


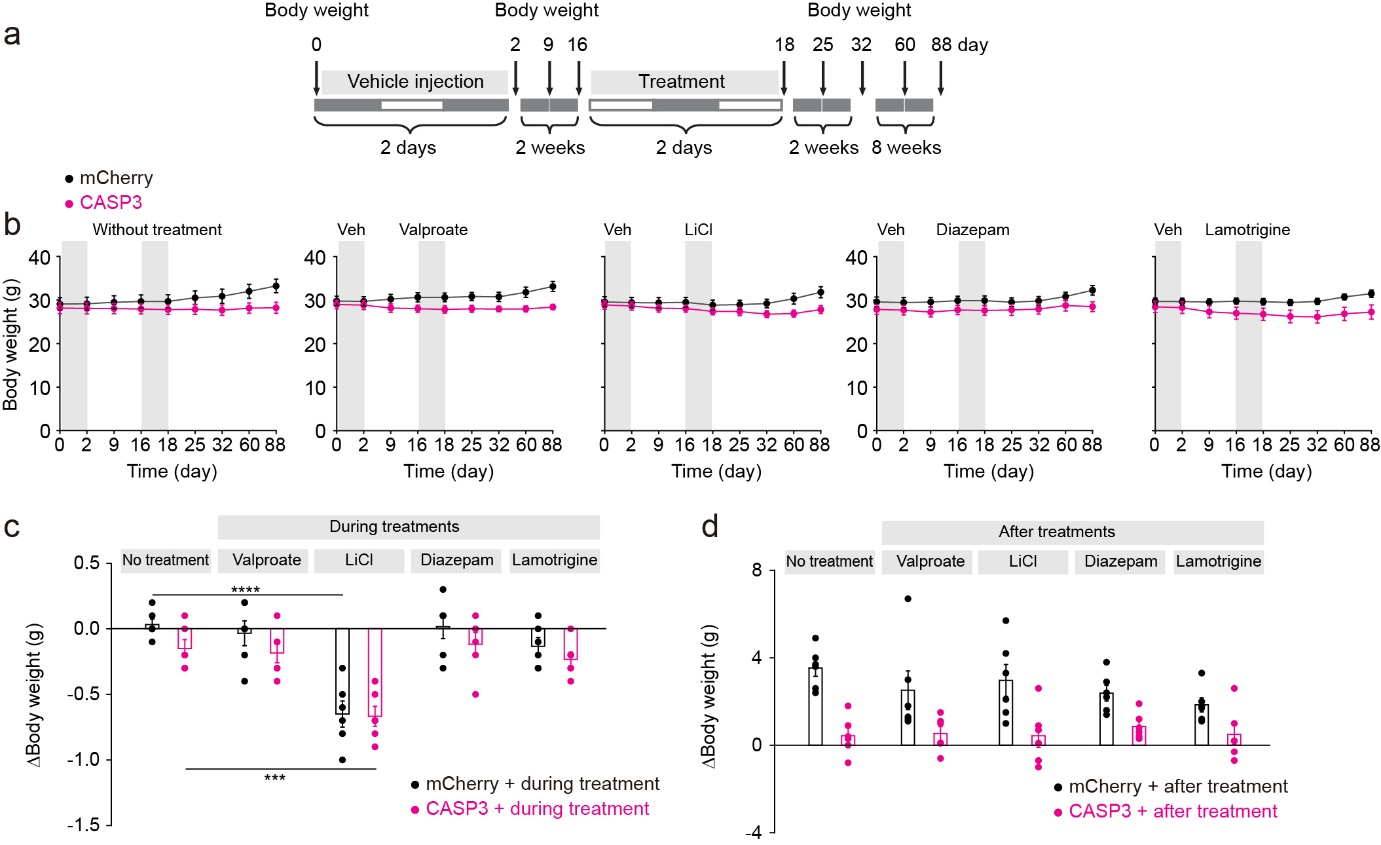


**Supplementary Figure 7 (supports Figure 3)**

**Body weight of the mice during and after treatments**.

1. Measurement of body weight for all drug treatments. The top arrows indicate body weight measurement.
2. Body weight of VTA*^Vgat^*-mCherry mice or VTA*^Vgat^-*CASP3 mice that received either vehicle or drug treatments or had no treatments.
3. Change of body weight during the 2-day treatments or without treatments. Two-way ANOVA and Bonferroni-Holm *post hoc* test. mCherry: no treatment vs. LiCl t(10)=6.34, ****p=0.00008. CASP3: no treatment vs. LiCl t(10)=5, ***p=0.0004.
4. Change of body weight after treatments or without treatment.


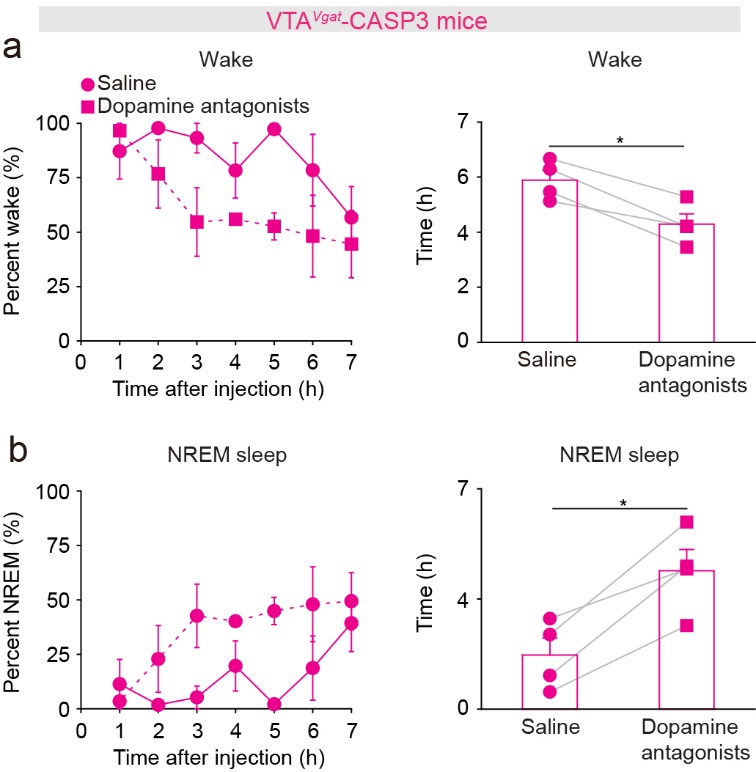


**Supplementary Figure 8 (supports Figure 5)**

**Blocking dopamine signaling restores extended wakefulness of VTA*^Vgat^*-CASP3 mice**

1. Percentage wake of VTA*^Vgat^*-CASP3 mice (n=4 mice) that received injections of saline or a dopamine receptor antagonist mixture (SCH-23390 and raclopride for D1 and D2/D3 receptors, respectively). Paired t-test, t(3)=5.75, *p=0.01.
2. Percentage NREM sleep of VTA*^Vgat^*-CASP3 mice that received either saline or the dopamine receptor antagonist mixture. Paired t-test, t(3)=-5.39, *p=0.01.


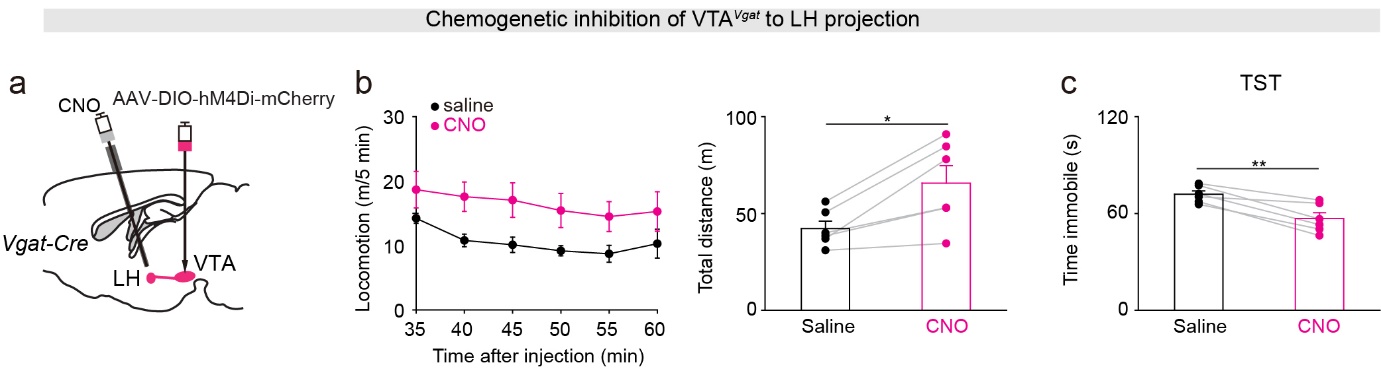


**Supplementary Figure 9 (supports Figure 5)**

**Chemogenetic inhibition of the VTA*^Vgat^* to LH projection produces hyperlocomotion and less depressive-like behavior**

1. AAV-DIO-hM4Di-mCherry was injected into the VTA of *Vgat-cre* mice. A guided cannula to deliver CNO (1mM) or saline was implanted above the LH.
2. Locomotion speed and distance travelled for VTA*^Vgat^-*hM4Di mice (n=6 mice) that received either saline or CNO into the LH through the cannula. Paired t-test, t(5)=-3.8, *p=0.01.
3. Immobility time during the tail-suspension test (TST) for VTA*^Vgat^-*hM4Di mice (n=6 mice) that received either saline or CNO injection into the LH. Paired t-test, t(5)=5.42, **p=0.002.
